# Diagnostic accuracy of Ascertain Dementia 8-item Questionnaire by participant and informant–A systematic review and meta-analysis

**DOI:** 10.1371/journal.pone.0291291

**Published:** 2023-09-12

**Authors:** Rajiv Tanwani, Mercy O. Danquah, Nina Butris, Aparna Saripella, Ellene Yan, Paras Kapoor, Marina Englesakis, David F. Tang-Wai, Maria Carmela Tartaglia, David He, Frances Chung

**Affiliations:** 1 Temerty Faculty of Medicine, University of Toronto, Toronto, Ontario, Canada; 2 Department of Anesthesia and Pain Management, Toronto Western Hospital, University Health Network, Toronto, Ontario, Canada; 3 Library & Information Services, University Health Network, Toronto, Ontario, Canada; 4 Division of Neurology, Department of Medicine, University of Toronto, Toronto, Ontario, Canada; 5 Department of Anesthesiology and Pain Medicine, Mount Sinai Hospital, Sinai Health, Toronto, Ontario, Canada; Clinical Investigation Center, TUNISIA

## Abstract

**Background:**

The Ascertain Dementia 8-item Questionnaire (AD8) is a screening tool for cognitive impairment that can be administered to older persons and/or their informants.

**Objectives:**

To evaluate the diagnostic accuracy and compare the predictive parameters of the informant and participant-completed Ascertain Dementia 8-item Questionnaire (iAD8 and pAD8, respectively) in older adults with cognitive impairment.

**Methods/Design:**

We searched ten electronic databases (including MEDLINE (Ovid), Embase) from tool inception to March 2022. We included studies with patients ≥60 years old that were screened for cognitive impairment using AD8 in any healthcare setting. Predictive parameters were assessed against reference standards to estimate accuracy and diagnostic ability using bivariate random-effects meta-analyses. We used QUADAS-2 criteria to assess risk of bias.

**Results:**

A cut-off of ≥2/8 was used to classify mild cognitive impairment (MCI), dementia, and cognitive impairment (MCI or dementia). Seven studies using the iAD8 (n = 794) showed a sensitivity of 80% and specificity of 79% to detect MCI. Nine studies using the iAD8 (n = 2393) established 91% sensitivity and 64% specificity to detect dementia. To detect MCI using the pAD8, four studies (n = 836) showed 57% sensitivity and 71% specificity. To detect dementia using the pAD8, four studies (n = 3015) demonstrated 82% sensitivity and 75% specificity. Recurring high or unclear risk of bias was noted in the domains of “Index test” and “reference standard”.

**Conclusions:**

The diagnostic accuracy of iAD8 is superior to that of pAD8 when screening for cognitive impairment. The AD8 may be an acceptable alternative to screen for cognitive impairment in older adults when there are limitations to formal testing.

## 1. Introduction

Over 55 million people in the world live with dementia—a syndrome marked by a progressive cognitive decline impacting normal functioning [1]. The World Health Organization estimates that the global prevalence of dementia will increase to 78 million by 2030 and 139 million by 2050 [2]. Many older adults develop a dementia precursor known as mild cognitive impairment (MCI), with prevalence ranging from 6.7% to 25.2% [3]. Contrary to those with dementia, individuals with MCI develop cognitive deficits, unexplained by normal aging or education level, that do not impact their activities of daily living and function [4].

Given the rising prevalence of cognitive impairment (CI) in older adults and the lack of adequate reporting of cognitive symptoms to physicians, it is imperative to have screening tools to detect CI among different populations [5]. Although cognitive screening tools are insufficient for diagnosis, they are time efficient and allow for appropriate referral to specialists to provide diagnosis and early management. Screening tools are especially helpful in environments such as primary care, preoperative clinic, and emergency departments, where both time and resources are limited and do not allow for a comprehensive assessment [6].

The Ascertain Dementia 8-item Questionnaire (AD8) is a brief screening tool for detecting CI [7]. It was developed using the Clinical Dementia Rating (CDR), a gold-standard informant-based scale commonly used in research settings to diagnose and stage dementia severity [8]. The AD8 consists of eight ‘Yes/No’ questions that evaluate various cognitive domains such as memory, orientation, judgement, and function, and are answered by informants [7]. The eight questions are: 1) problems with judgement (e.g. problems making decisions, bad financial decisions, problems with thinking); 2) less interest in hobbies/activities; 3) repeats the same things over and over (questions, stories, or statements); 4) trouble learning how to use a tool, appliance, or gadget (e.g. VCR, computer, microwave, remote control); 5) forgets correct month or year; 6) trouble handling complicated financial affairs (e.g. balancing check book, income taxes, paying bills); 7) trouble remembering appointments; and 8) daily problems with thinking and/or memory [7].

A cut-off score of two or greater suggests that a patient may have CI and should undergo further evaluation and diagnosis [7]. AD8 has been recommended by a subgroup of the fifth Canadian Consensus Conference on the Diagnosis and Treatment of Dementia as a potential informant-based tool to assess the cognition and function of patients suspected of neurocognitive disorders in clinical settings [9]. The AD8 has also been validated for use as a self-completed CI screening tool [10]. A self-rated CI instrument may be particularly useful in situations where there is an absence of reliable informants or a limitation on patient caretakers in the hospital such as during the COVID-19 pandemic [10, 11].

This systematic review and meta-analysis aims to evaluate the diagnostic accuracy and qualitatively compare the predictive parameters of AD8 by informant (iAD8) and/or participant (pAD8) for screening CI in all healthcare settings.

## 2. Methods

### 2.1 Study registration

The study protocol was registered in the International Prospective Register of Systematic Reviews (PROSPERO) [CRD 42022331798]. We used the Preferred Reporting Items for Systematic Reviews and Meta-Analyses (PRISMA) guidelines to report this systematic review [12].

### 2.2 Search strategy

Systematic, structured literature searching was conducted by an information specialist (ME). All searches were conducted on March 28, 2022, using the Ovid platform for the following databases: MEDLINE (Ovid), MEDLINE In-Process/ePubs, Embase, Cochrane Central Register of Controlled Trials, Cochrane Database of Systematic Reviews, APA PsycINFO, Ovid Emcare Nursing and Journals@Ovid; Cumulative Index to Nursing and Allied Health Literature (CINAHL, EbscoHost), Web of Science (Clarivate Analytics), and Scopus (Elsevier). Preliminary searches were conducted, and full-text literature was mined for potential keywords and appropriate controlled vocabulary terms (such as Medical Subject Headings for MEDLINE and EMTREE descriptors for Embase). The Yale MeSH Analyzer was used to assess target citations [13]. The search strategy concept blocks were built on the topics of: ("Ascertain Dementia 8" OR “AD8”), AND ("dementia", OR “cognition”, OR “psychometrics”), with each component being fleshed out with controlled vocabularies, text word terms, and synonyms. Results were limited to the English language, humans, adults ≥60 years, and literature published from 2004 to the search date. A citation search of 3 key articles from the creators of the AD8 tool was completed on the Web of Science and Scopus databases [7, 10, 14].

Supplemental searching was conducted by team members (RT, MD) using citation search via Google Scholar, and PubMed. The reference lists of included studies were examined to identify any additional articles missed in the initial search and continued literature surveillance was conducted throughout the year. The complete detailed search strategy is included in the supplement.

### 2.3 Study inclusion criteria

The study inclusion criteria were: 1) patients with an average age ≥ 60 years screened for CI (i.e. MCI or dementia) using iAD8 and/or pAD8; 2) AD8 used in all healthcare settings such as community, primary care, secondary care, memory clinics, hospitalized patients, emergency departments; 3) comparison with clinical diagnosis using reference standards such as Diagnostic and Statistical Manual of Mental Disorders (DSM) III-V criteria for dementia, Petersen’s criteria for MCI, CDR, neuropsychological test battery, or panel consensus; 4) reporting of predictive parameters: sensitivity, specificity, positive predictive value (PPV), negative predictive value (NPV), area under the curve (AUC), positive likelihood ratio (PLR), negative likelihood ratio (NLR); and 5) randomized controlled trials, cohort studies (retrospective and prospective), case-control, cross-sectional, and observational studies. The exclusion criterion was non-English studies.

### 2.4 Study selection and data extraction

All identified articles from the electronic databases were uploaded onto Covidence, an online systematic review manager tool [15]. After duplicate removal, two reviewers (RT, MD) independently performed title, abstract, and full-text screening using the defined inclusion and exclusion criteria. After full-text screening, data extraction was completed independently by the same reviewers (RT, MD). All conflicts were resolved through discussion with a third reviewer (AS). The following information was extracted from the included studies: demographics, country, year completed, study design, and predictive parameters of cognitive screening.

### 2.5 Statistical analysis

We performed meta-analysis using RevMan Review Manager version 5.4 and Meta disc version 1.4. AD8 cut-off of two or greater was used as the threshold for having MCI, dementia, and CI (either MCI or dementia). We reconstructed a 2x2 contingency table for each outcome at a cut-off of two or greater. The results from individual studies of iAD8 and pAD8 were combined using a random-effects bivariate analysis model to obtain the summary estimates with a 95% confidence interval. Paired outcomes such as the sensitivity and specificity of each study were analyzed using this method. The following test characteristics were recalculated with 95% confidence interval: prevalence, sensitivity, specificity, PPV, NPV, PLR, NLR, log scale diagnostic odds ratio (DOR), and AUC. Forest plots were created using a random-effects model. We presented the logscale DOR and AUC to evaluate the diagnostic ability of the informant and participant completed AD8. The test characteristics of primary interest include sensitivity, specificity, and AUC. Heterogeneity (I^2^) was explored using the χ2 test and a P-value <0.05 was considered statistically significant. We performed the leave-one-study-out meta-analysis to assess the effect of each study on the combined estimates of sensitivity, specificity, log scale DOR, and heterogeneity. Meta-regression was performed using Open Meta Analyst for the variables age, sample size, and female gender when the number of studies was five or greater [16]. This was done to evaluate the association of variables with the combined estimates of sensitivity, specificity, and log scale DOR. We reported the values at other cut-offs in a tabular manner in the supplement.

### 2.6 Assessment of study quality

We used the Quality Assessment of Diagnostic Accuracy Studies (QUADAS-2) for the quality assessment of the included studies [17]. Two reviewers (EY, PK) independently and critically appraised each included study. All conflicts were resolved by discussion with a third author (AS). The QUADAS-2 assessed the studies across four domains (patient selection, index test, reference standard, and flow and timing), and each domain was further assessed in terms of risk of bias and applicability. Signalling questions were included to facilitate the risk of bias assessment based on the responses provided (these were answered as “yes,” “no,” or “unclear”, and “yes” indicates a low risk of bias). We calculated the interrater agreement percentage and Cohen’s Kappa value for the overall risk of bias.

## 3. Results

### 3.1 Study selection and characteristics

**Fig 1:**
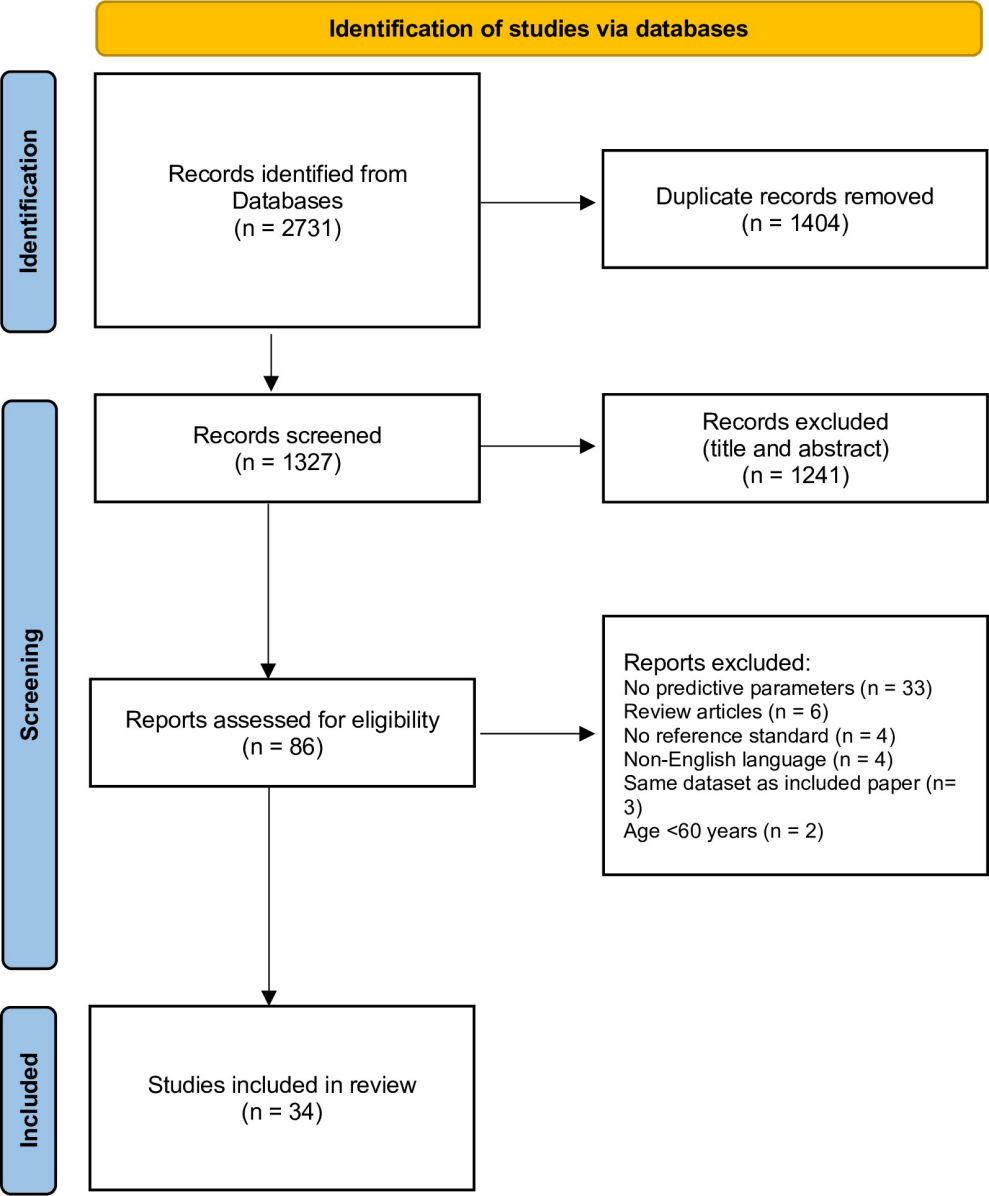
PRISMA study flow diagram.

Our literature search resulted in 2,731 articles (Fig 1). After removal of duplicates, 1,327 articles remained. Following a review of titles and abstracts, 1,241 articles were excluded. Of the 86 full-text articles assessed for eligibility, 34 articles with 24,778 participants (mean age 74.3 ± 7.6 years, 54.3% female) were included (Fig 1). Nineteen were cross-sectional studies, six were case-control studies, eight were prospective cohort studies, and one was a retrospective cohort study. The countries of origin included Brazil (n = 1), China (n = 4), India (n = 1), Japan (n = 1), Korea (n = 2), Lebanon (n = 1), Philippines (n = 1), Singapore (n = 5), Spain (n = 1), Taiwan (n = 4), Turkey (n = 1), United Kingdom (n = 4), and the United States (n = 8). Of these articles, CI was mainly diagnosed using CDR (n = 13), more than one reference standard (n = 10), and the DSM (III-V) (n = 6) (Tables 1 and 2).

**Table 1:** Demographics and study characteristics of included studies in community/primary care settings.

Author, year country	Study design	Patient population	No. (N) of patients	Age (years) mean ± SD	Gender female–n (%)	Reference standard(s) used
**Cai 2021** **China [18]**	Cross-sectional	Community	523	68.0 ± 9.7	289 (55.3)	MCI: Petersen’s criteria Dem: DSM-IV
**Chan 2016** **Singapore [19]**	Cross-sectional	Primary care	309	71.7 ± 8.2	168 (54.3)	CDR
**Chen 2018** **Taiwan [20]**	Retrospective cohort	Community	40	AD8 ≥2: 67.1 ± 10.8 AD8 <2: 63.1 ± 10.0	34 (85)	Dem: consensus panel of specialists
**Chin 2013** **Singapore [21]**	Case-control	NC: Community MCI/AD: memory/dem clinic	178	66.7 ± 10.8	119 (66.9)	MCI: Petersen’s criteria AD: NINCDS-ADRDA criteria
**Correia 2011** **Brazil [22]**	Cross-sectional	Community	109	Median age: 76.7 ± 0.6	87 (79.8)	DSM-IV CDR
**Dominguez 2021 Philippines [23]**	Cross-sectional	Community	366	73.4 ± 7.0	256 (69.9)	DSM-IV TR
**Dong 2021** **China [24]**	Cross-sectional	Community	4481 DTA: 4463	71.1 ± 4.9	2545 (56.8)	DSM-IV
**Galvin 2010** **US [25]**	Prospective cohort	Community	257	75.4 ± 7.3	140 (54.5)	CDR
**Galvin 2007** **US [10]**	Prospective cohort	Community	325	76.8 ± 8.9	185 (57)	CDR
**Galvin 2005** **US [7]**	Prospective cohort	Community	236	78.1 ± 9.2	125 (53)	CDR
**Kan 2019** **Singapore [26]**	Cross-sectional	Community	761	CDR 0: 68.7 ± 6.0 CDR 0.5: 72.7 ± 6.8 CDR 1: 78.3 ± 5.8	409 (53.7)	CDR
**Kasai 2021** **Japan [27]**	Cross-sectional	Community	93	CDR 0: 80.0 ± 4.1 CDR 0.5: 80.2 ± 4.0 CDR ≥1: 83.8 ± 3.4	58 (62.4)	CDR
**Kim 2019** **Korea [28]**	Cross-sectional	Community	420	75.3 ± 6.0	233 (55.5)	MCI: Petersen’s criteria Dem: DSM-IV
**Malmstrom 2009** **US [29]**	Prospective cohort	Community	61	61.3 ± 4.4	39 (63.9)	CDR
**Mao 2018** **Taiwan [30]**	Cross-sectional	Community	10360 DTA: 8805	74.9 ± 6.0	5422 (52.3)	Dem: NIA-AA criteria
**Morris 2020** **UK [31]**	Prospective cohort	Community	230	80.4 (no SD) NC: 81.6 ± 9.2 AD: 79.6 ± 8.5	111 (48.2)	Neuropathological AD Non-neuropathologic AD: NIA-Reagan Institute criteria
**Shang 2021** **China [32]**	Cross-sectional	Community (military personnel)	160	80.7 ± 7.5	5 (3.1)	CI: DSM-V
**Yang 2016** **China [33]**	Cross-sectional	Community	2015	79.5 ± 7.6	1165 (57.8)	Dem: NIA-AA criteria
**Yang 2011** **Taiwan [34]**	Case-control	Community	239	CDR 0: 72.5 ± 5.7 CDR 0.5: 75.1 ± 8.4 CDR 1: 75.4 ± 9.6	129 (53.9)	CDR

Abbreviations: AD, Alzheimer’s Dementia; CDR, Clinical Dementia Rating; CI, Cognitive Impairment; Dem, Dementia; DSM, Diagnostic and Statistical Manual of Mental Disorders; DTA, Diagnostic Test Accuracy; MCI, Mild Cognitive Impairment; NC, Normal Cognition; NIA-AA, National Institute on Aging and Alzheimer’s Association; NINCDS-ADRDA, National Institute of Neurological and Communicative Disorders and Stroke and the Alzheimer’s Disease and Related Disorders Association.

**Table 2:** Demographics and study characteristics of included studies in secondary care/memory clinic and tertiary care/hospitalized settings.

Author, year country	Study design	Patient population	No. (N) of patients	Age (years) mean ± SD	Gender female–n (%)	Reference standard(s) used
***Secondary care/memory clinic setting***						
**Carnero Pardo 2013** **Spain [35]**	Cross-sectional	Neurology unit	407	74.8 ± 9.0	247 (60.7)	MCI: SEN study group Dem: DSM-IV
**Chio 2018** **Taiwan [36]**	Cross-sectional	Neurology clinic	153	76.9 ± 8.6	100 (65.4)	CDR
**Dong 2013** **Singapore [37]**	Case-control	Memory clinic	280	73.4 ± 8.6	152 (54.3)	CDR
**Galvin 2006** **US [14]**	Cross-sectional	Memory clinic	255	73.3 ± 11.3	141 (56.4)	CDR
**Karam 2018** **Lebanon [38]**	Case-control	Out-pt clinic, in-pt unit, nursing home	132	81.9 ± 7.8	81 (61.4)	NINCDS-ADRDA
**Larner 2017** **UK [39]**	Cross-sectional	Memory clinic	46	62.0 ± 12.7	19 (41.3)	MCI: Petersen’s criteria Dem: DSM-IV-TR
**Mansbach 2016** **US [40]**	Prospective cohort	Long-term care	357	82.3 ± 9.2	228 (63.9)	MCI: Petersen’s criteria Dem: DSM-IV CDR
**Razavi 2014** **US [41]**	Cross-sectional	Neurology/memory clinic	186	77.8 ± 8.2	125 (67.2)	MCI: Petersen’s criteria Dem: various criteria based on sub-type
**Ryu 2009** **Korea [42]**	Case-control	Memory/dem clinic	155	69.5 ± 8.1	112 (72.3)	CDR
**Tak 2021^†^** **India [43]**	Cross-sectional	Secondary care/out-pt	776	65–74: 583 75–84: 180 ≥85: 13	200 (25.8)	ICD-10
**Tew 2015** **Singapore [44]**	Case-control	Memory clinic	245	NC: 67.9 ± 7.0 Dem: 76.6 ± 8.1	164 (66.9)	Dem: DSM-IV and various criteria based on sub-type
**Usarel 2019** **Turkey [45]**	Cross-sectional	Memory clinic	334	74.5 ± 8.5	217 (65.7)	MCI: DSM-V Dem: DSM-V
***Tertiary care/hospitalized setting***						
**Duggan 2020** **US [46]**	Cross-sectional	ICU	75	63.7 ± 17.8	43 (57)	CDR
**Jackson 2016** **UK [47]**	Prospective cohort	Hospital pt with delirium	77	84.4 ± 6.7	53 (68.8)	DSM-IV
**Taylor-Rowan** **2022 UK [48]**	Prospective cohort	Tertiary-care	137 DTA: 102	69.7 ± 15.0	51/134 (38.1)	MCI: DSM-V Dem: DSM-V

Abbreviations: CDR, Clinical Dementia Rating; Dem, Dementia; DSM, Diagnostic and Statistical Manual of Mental Disorders; DTA, Diagnostic Test Accuracy; ICD-10, International Statistical Classification of Diseases and Related Health Problems 10^th^ Revision; In-pt, In-patient; MCI, Mild Cognitive Impairment; NC, Normal Cognition; NINCDS-ADRDA, National Institute of Neurological and Communicative Disorders and Stroke and the Alzheimer’s Disease and Related Disorders Association; Out-pt, Out-patient; TIA, Transient Ischemic Attack.

^†^Ages reported in range

The included studies were divided into three settings: 1) community/primary care (n = 19) [7, 10, 18–34], 2) secondary care/memory clinic (n = 12) [14, 35–45], and 3) tertiary care/hospitalized (n = 3) [46–48] (Tables 1 and 2). No studies were conducted in the perioperative setting such as preoperative clinics. In comparison to normal cognition, the studies were divided into three subgroups: MCI, dementia, and cognitive impairment (MCI or dementia).

### 3.2 Classification of included studies based on healthcare setting

In the community/primary care setting, 19 studies with 21,163 patients were included. Of these, 11 were cross-sectional studies [18, 19, 22–24, 26–28, 30, 32, 33], five were prospective cohort studies [7, 10, 25, 29, 31], two were case-control studies [21, 34], and one was a retrospective cohort study [20]. The mean age was 74.2 ± 7.0 years old, and 54.4% were female.

In the secondary care/memory clinic setting, 12 studies with 3,326 patients were included. There were seven cross-sectional studies [14, 35, 36, 39, 41, 43, 45], four case-control studies [37, 38, 42, 44], and one prospective cohort study [40]. The mean age was 75.5 ± 9.9 years old, and 53.8% were female.

In the tertiary care/hospitalized setting, three studies with 289 patients were included, of which two were prospective cohort studies [47, 48], and one was cross-sectional [46]. The mean age was 72.0 ± 16.1 years old, and 50.9% were female.

### 3.3 Pooled predictive parameters of iAD8 and pAD8

The pooled predictive parameters of the iAD8 and pAD8 were calculated based on degree of CI (Table 3). This analysis was also completed for each tool separately based on study setting (Table 4).

**Table 3:** Pooled predictive parameters of informant and participant completed AD8 (cut off 2 or greater).

**iAD8 ≥2**			
**Predictive parameters**	**MCI vs control**	**Dementia vs control**	**CI vs control**
	(7 studies, n = 794)	(9 studies, n = 2,393)	(11 studies, n = 2,021)
Prevalence	0.42 (0.39–0.45)	0.36 (0.33–0.37)	0.66 (0.64–0.68)
Sensitivity	0.80 (0.75–0.84)	0.91 (0.89–0.93)	0.88 (0.86–0.90)
Specificity	0.79 (0.75–0.83)	0.64 (0.62–0.67)	0.75 (0.72–0.79)
PPV	0.73 (0.68–0.77)	0.58 (0.55–0.61)	0.87 (0.86–0.89)
NPV	0.84 (0.81–0.88)	0.93 (0.91–0.94)	0.77 (0.73–0.80)
DOR	18.96 (9.21–39.05)	24.44 (6.70–89.17)	18.81 (8.20–43.17)
SROC	AUC = 0.8838 SE = 0.0286	AUC = 0.7970 SE = 0.0999	AUC = 0.9086 SE = 0.0298
**pAD8 ≥2**			
**Predictive parameters**	**MCI vs control**	**Dementia vs control**	**CI vs control**
	(4 studies, n = 836)	(4 studies, n = 3,105)	(4 studies, n = 936)
Prevalence	0.42 (0.39–0.45)	0.28 (0.26–0.29)	0.68 (0.65–0.71)
Sensitivity	0.57 (0.52–0.62)	0.82 (0.79–0.84)	0.62 (0.58–0.65)
Specificity	0.71 (0.67–0.75)	0.75 (0.73–0.77)	0.72 (0.67–0.77)
PPV	0.59 (0.53–0.64)	0.55 (0.53–0.58)	0.82 (0.78–0.86)
NPV	0.70 (0.65–0.74)	0.92 (0.90–0.93)	0.47 (0.43–0.52)
DOR	2.86 (1.9–4.31)	10.70 (2.14–53.54)	4.06 (2.15–7.68)
SROC	AUC = 0.6698 SE = 0.0478	AUC = 0.7930 SE = 0.1707	AUC = 0.6222 SE = 0.2251

Abbreviations: CI, cognitive impairment; DOR, diagnostic odds ratio; iAD8, informant completed AD8; MCI, mild cognitive impairment; NPV, negative predictive value; pAD8, participant completed AD8; PPV, positive predictive value; SROC, summary receiver operating characteristic

**Table 4:** Pooled predictive parameters of informant and participant completed AD8 (cut off 2 or greater) based on study setting.

**iAD8 ≥2**									
	**Community/primary care**			**Secondary care/memory clinic**			**Tertiary care/hospitalized**		
**Predictive parameters**	**MCI vs control**	**Dementia vs control**	**CI vs control**	**MCI vs control**	**Dementia vs control**	**CI vs control**	**MCI vs control**	**Dementia vs control**	**CI vs control**
	(4 studies, n = 645)	(4 studies, n = 1,273)	(4 studies, n = 960)	(3 studies, n = 621)	(3 studies, n = 1,094)	(5 studies, n = 884)	NA	(2 studies, n = 177)	(2 studies, n = 177)
Prevalence	0.28 (0.25–0.32)	0.43 (0.40–0.46)	0.51 (0.48–0.54)	0.24 (0.21–0.28)	0.23 (0.20–0.26)	0.82 (0.79–0.84)	NA	0.28 (0.21–0.35)	0.64 (0.57–0.71)
Sensitivity	0.82 (0.76–0.87)	0.92 (0.89–0.94)	0.86 (0.82–0.89)	0.77 (0.69–0.83)	0.87 (0.82–0.91)	0.90 (0.87–0.92)	NA	0.96 (0.86–1.00)	0.89 (0.82–0.94)
Specificity	0.78 (0.74–0.81)	0.67 (0.64–0.70)	0.81 (0.77–0.84)	0.84 (0.80–0.87)	0.64 (0.61–0.68)	0.62 (0.54–0.69)	NA	0.45 (0.36–0.54)	0.65 (0.52–0.77)
PPV	0.59 (0.53–0.65)	0.67 (0.64–0.71)	0.83 (0.79–0.86)	0.61 (0.53–0.67)	0.42 (0.38–0.46)	0.91 (0.89–0.93)	NA	0.40 (0.31–0.50)	0.82 (0.74–0.88)
NPV	0.92 (0.86–0.94)	0.92 (0.89–0.94)	0.84 (0.81–0.87)	0.92 (0.89–0.94)	0.94 (0.92–0.96)	0.57 (0.49–0.64)	NA	0.96 (0.87–0.99)	0.77 (0.63–0.87)
DOR	16.32 (10.51–25.34)	22.59 (16.01–31.88)	25.68 (18.23–36.17)	17.08 (10.88–26.81)	12.32 (8.28–18.32)	14.15 (9.5–21.08)	NA	19.47 (4.53–83.61)	15.84 (7.18–34.95)
SROC	AUC = 0.8539 SE = 0.0570	AUC = 0.9437 SE = 0.0592	AUC = 0.9104 SE = 0.0620	AUC = 0.9204 SE = 0.0210	AUC = 0.7259 SE = 0.0395	AUC = 0.9187 SE = 0.0348	NA	NA	NA
**pAD8 ≥2**									
	**Community/primary care**			**Secondary care/memory clinic**			**Tertiary care/hospitalized**		
**Predictive parameters**	**MCI vs control**	**Dementia vs control**	**CI vs control**	**MCI vs control**	**Dementia vs control**	**CI vs control**	**MCI vs control**	**Dementia vs control**	**CI vs control**
	(2 studies, n = 701)	(3 studies, n = 2,958)	(2 studies, n = 503)	(2 studies, n = 637)	NA	(2 studies, n = 433)	NA	NA	NA
Prevalence	0.27 (0.23–0.30)	0.21 (0.20–0.23)	0.56 (0.51–0.60)	0.26 (0.22–0.29)	NA	0.82 (0.77–0.85)	NA	NA	NA
Sensitivity	0.51 (0.44–0.59)	0.85 (0.81–0.88)	0.66 (0.60–0.71)	0.64 (0.56–0.71)	NA	0.58 (0.53–0.63)	NA	NA	NA
Specificity	0.74 (0.70–0.77)	0.77 (0.75–0.78)	0.75 (0.69–0.81)	0.58 (0.54–0.63)	NA	0.64 (0.52–0.74)	NA	NA	NA
PPV	0.42 (0.35–0.48)	0.50 (0.47–0.53)	0.77 (0.71–0.82)	0.35 (0.29–0.40)	NA	0.87 (0.83–0.91)	NA	NA	NA
NPV	0.80 (0.77–0.84)	0.95 (0.94–0.96)	0.63 (0.58–0.70)	0.82 (0.78–0.86)	NA	0.26 (0.20–0.32)	NA	NA	NA
DOR	2.96 (2.09–4.19)	18.5 (14.58–23.48)	5.94 (4.02–8.8)	2.48 (1.72–3.58)	NA	2.44 (1.47–4.03)	NA	NA	NA
SROC	NA	AUC = 0.8254 SE = 0.0184	NA	NA	NA	NA	NA	NA	NA

Abbreviations: CI, cognitive impairment; DOR, diagnostic odds ratio; iAD8, informant completed AD8; MCI, mild cognitive impairment; NA, not available; NPV, negative predictive value; pAD8, participant completed AD8; PPV, positive predictive value; SROC, summary receiver operating characteristic

### 3.4 Pooled predictive parameters of iAD8 to detect MCI

**Fig 2:**
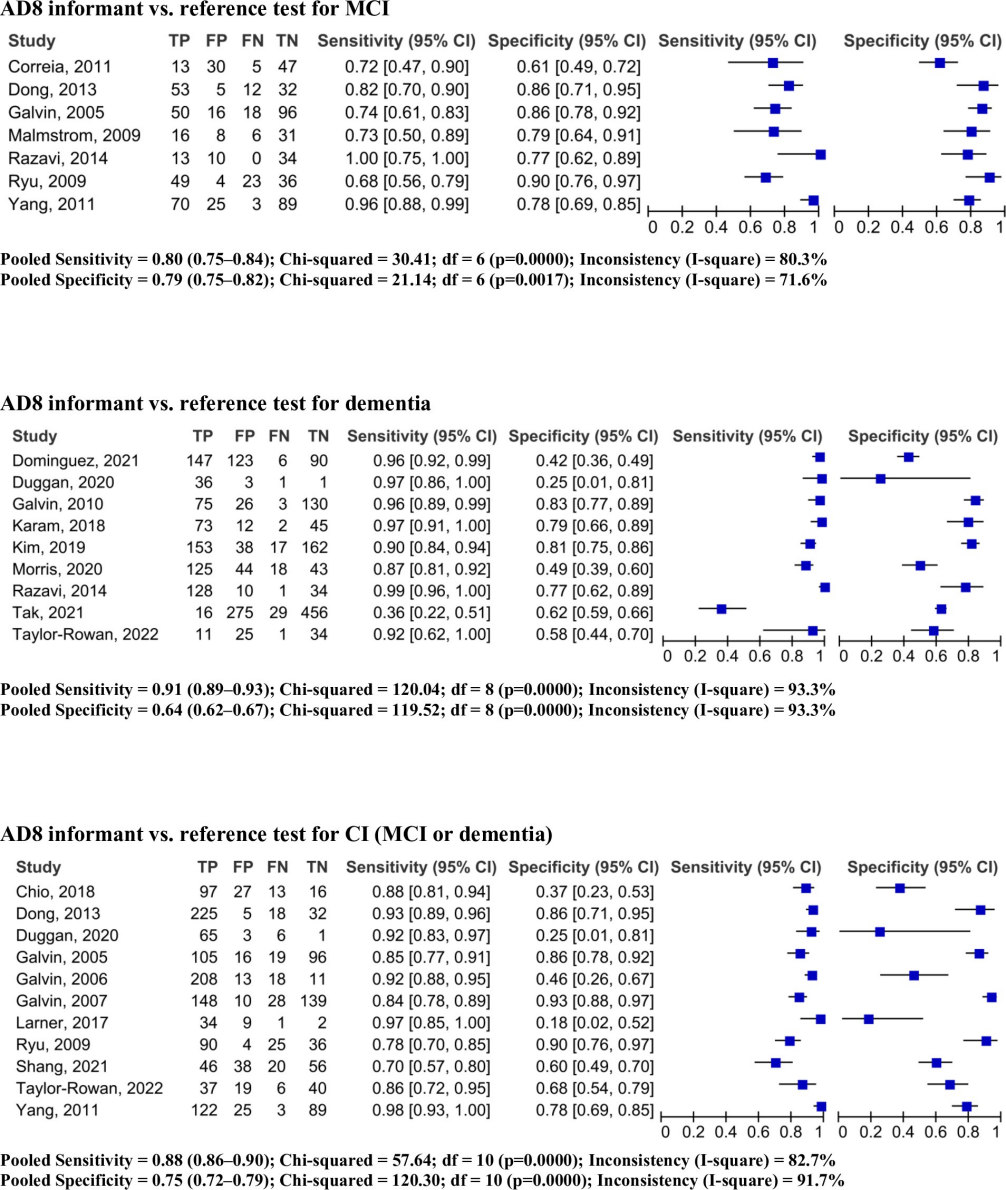
Pooled forest plots of sensitivity and specificity of informant completed AD8 (cut off 2 or greater). TP, true positive; FP, false positive; FN, false negative; TN, true negative.

**Fig 3:**
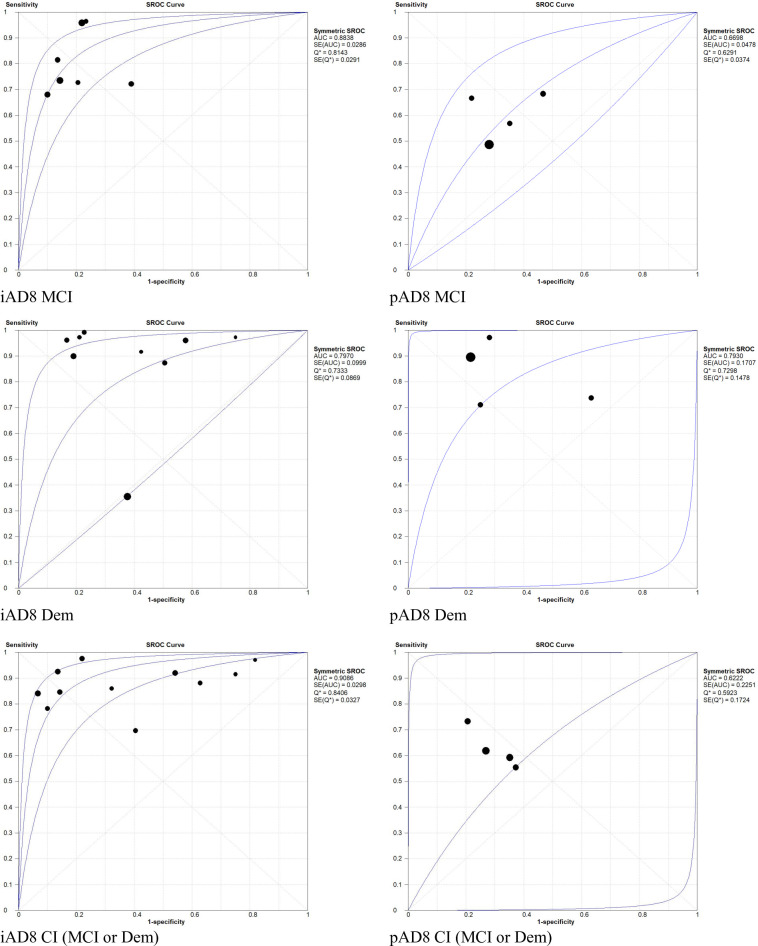
Summary receiver operating characteristic of the iAD8 and pAD8. AUC, area under the curve; Dem, Dementia; MCI, mild cognitive impairment; Q*, point of indifference on the ROC curve; SE, standard error; SROC, summary receiver operating characteristic.

The iAD8 was used in seven studies (794 participants) at a cut-off of two to detect MCI [7, 22, 29, 34, 37, 41, 42]. The overall prevalence of MCI as determined by the reference standard was 42% (95% CI: 39, 45%). The iAD8 showed 80% sensitivity (95% CI: 75, 84%, I^2^: 80.3%) and 79% specificity (95% CI: 75, 83%, I^2^: 71.6%). The AUC was 0.88, and the DOR was 18.96 (95% CI: 9.21, 39.05, I^2^: 60.9%) (Figs 2 and 3, Table 3).

### 3.5 Pooled predictive parameters of iAD8 to detect dementia

The iAD8 was used in nine studies (2,393 participants) at a cut-off of two to detect dementia [23, 25, 28, 31, 38, 41, 43, 46, 48]. The overall prevalence of dementia determined by the reference standard was 36% (95% CI: 33, 37%). The iAD8 showed 91% sensitivity (95% CI: 89, 93%, I^2^: 93.3%) and 64% specificity (95% CI: 62, 67%, I^2^: 93.3%). The AUC was 0.80, and the DOR was 24.44 (95% CI: 6.70, 89.17, I^2^: 93.2%) (Figs 2 and 3, Table 3).

### 3.6 Pooled predictive parameters of iAD8 to detect CI (MCI or dementia)

The iAD8 was used in 11 studies (2,021 participants) at a cut-off of two to detect CI (MCI or dementia) [7, 10, 14, 32, 34, 36, 37, 39, 42, 46, 48]. The overall prevalence of CI determined by the reference standard was 66% (95% CI: 64, 68%). The iAD8 achieved 88% sensitivity (95% CI: 86, 90%, I^2^: 82.7%) and 75% specificity (95% CI: 72, 79%, I^2^: 91.7%). The AUC was 0.91, and the DOR was 18.81 (95% CI: 8.20, 43.17, I^2^: 86.7%) (Figs 2 and 3, Table 3).

### 3.7 Pooled predictive parameters of pAD8 to detect MCI

**Fig 4:**
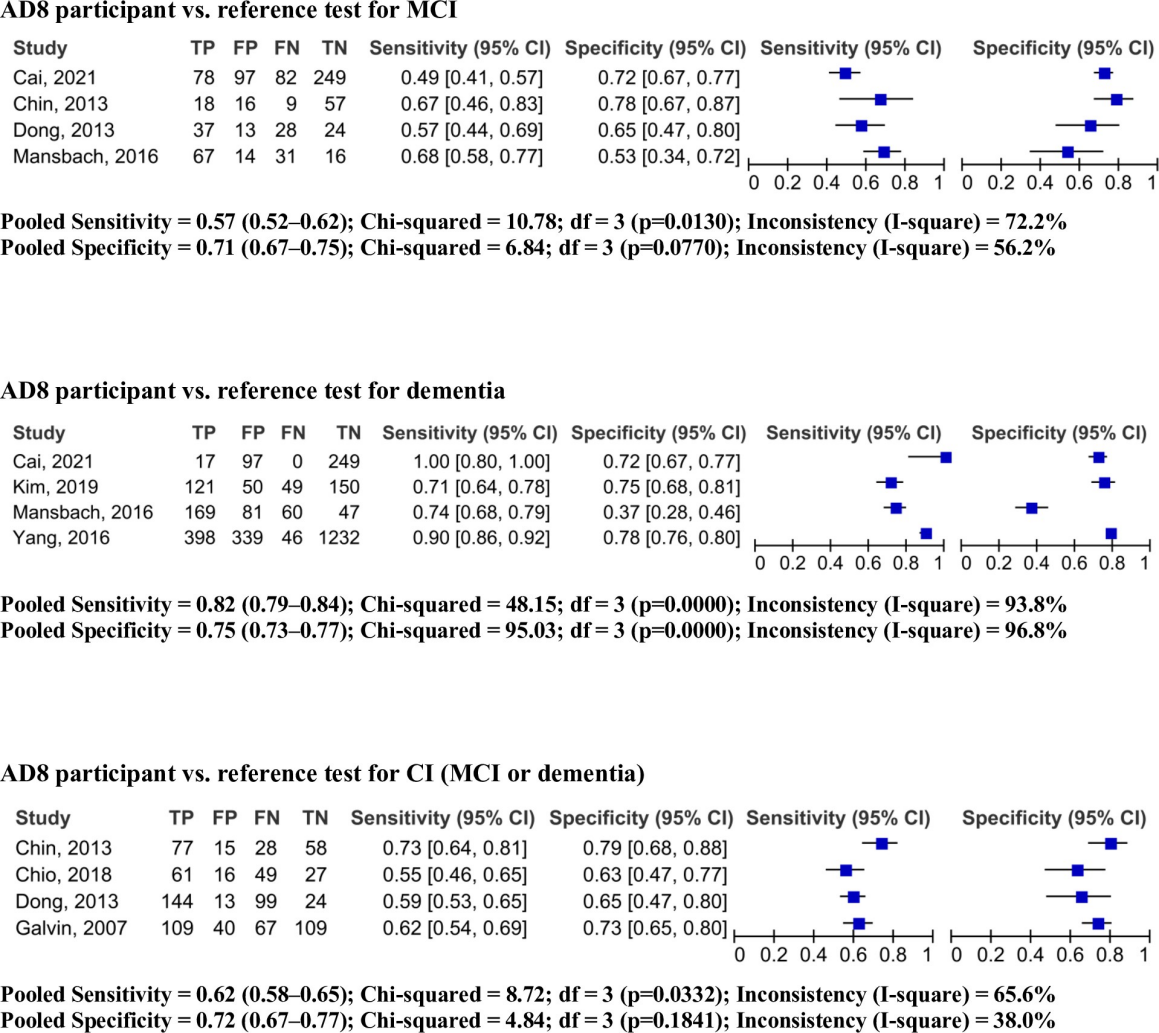
Pooled forest plots of sensitivity and specificity of participant completed AD8 (cut off 2 or greater). TP, true positive; FP, false positive; FN, false negative; TN, true negative.

The pAD8 was used in four studies (836 patients) at a cut-off of two to detect MCI [18, 21, 37, 40]. The overall prevalence of MCI determined by the reference standard was 42% (95% CI: 39, 45%). The pAD8 achieved 57% sensitivity (95% CI: 52, 62%, I^2^: 72.2%) and 71% specificity (95% CI: 67, 75%, I^2^: 56.2%). The AUC was 0.67, and the DOR was 2.86 (95% CI: 1.90, 4.31, I^2^: 28.1%) (Figs 3 and 4, Table 3).

### 3.8 Pooled predictive parameters of pAD8 to detect dementia

The pAD8 was used in four studies (3,105 patients) at a cut-off of two to detect dementia [18, 28, 33, 40]. The overall prevalence of dementia determined by the reference standard was 28% (95% CI: 26, 29%). The pAD8 achieved 82% sensitivity (95% CI: 79, 84%, I^2^: 93.8%) and 75% specificity (95% CI: 73, 77%, I^2^: 96.8%). The AUC was 0.79, and the DOR was 10.70 (95% CI: 2.14, 53.54, I^2^: 97.3%) (Figs 3 and 4, Table 3).

### 3.9 Pooled predictive parameters of pAD8 to detect CI (MCI or dementia)

The pAD8 was used in four studies (936 patients) at a cut-off of two to detect CI (MCI or dementia) [10, 21, 36, 37]. The overall combined prevalence of CI determined by the reference standard was 68% (95% CI: 65, 71%). The pAD8 showed 62% sensitivity (95% CI: 58, 65%, I^2^: 65.6%) and 72% specificity (95% CI: 67, 77%, I^2^: 38.0%). The AUC was 0.62, and the DOR was 4.06 (95% CI: 2.15, 7.68, I^2^: 74.0%) (Figs 3 and 4, Table 3).

### 3.10 Pooled predictive parameters of iAD8 and pAD8 in the community/primary care setting

The iAD8 was used in four studies (645 patients) at a cut-off of two to detect MCI [7, 22, 29, 34]. The overall prevalence of MCI determined by the reference standard was 28%. The iAD8 showed 82% sensitivity (95% CI: 76, 88, I^2^: 83.5%), 78% specificity (95% CI: 74, 82, I^2^: 84.2%), and the AUC was 0.85 (Table 4). The iAD8 was used in four studies (1,273 patients) to detect dementia [23, 25, 28, 31]. The overall prevalence of dementia determined by the reference standard was 43%. The iAD8 showed 92% sensitivity (95% CI: 89, 94, I^2^: 72.3%), 67% specificity (95% CI: 63, 70, I^2^: 97.4%), and the AUC was 0.94 (Table 4). The iAD8 was used in four studies (960 patients) to detect CI [7, 10, 32, 34]. The overall prevalence of CI determined by the reference standard was 51%. The iAD8 showed 86% sensitivity (95% CI: 82, 89, I^2^: 90.8%), 81% specificity (95% CI: 77, 84, I^2^: 93.1%), and the AUC was 0.91 (Table 4).

The pAD8 was used in two studies (701 patients) at a cut-off of two to detect MCI [18, 21]. The overall prevalence of MCI determined by the reference standard was 27%. The pAD8 showed 51% sensitivity (95% CI: 44, 59, I^2^: 66.9%) and 74% specificity (95% CI: 70, 77, I^2^: 54.5%) (Table 4). The pAD8 was used in three studies (2,958 patients) to detect dementia [18, 28, 33]. The overall prevalence of dementia determined by the reference standard was 21%. The pAD8 showed 85% sensitivity (95% CI: 82, 88, I^2^: 94.3%), 77% specificity (95% CI: 75, 78, I^2^: 78.4%), and the AUC was 0.83 (Table 4). The pAD8 was used in two studies (503 patients) to detect CI [10, 21]. The overall prevalence of CI determined by the reference standard was 56%. The pAD8 showed 66% sensitivity (95% CI: 60, 72, I^2^: 74.3%) and 75% specificity (95% CI: 69, 81, I^2^: 6.2%) (Table 4).

### 3.11 Pooled predictive parameters of iAD8 and pAD8 in the secondary care/memory clinic setting

The iAD8 was used in three studies (621 patients) at a cut-off of two to detect MCI [37, 41, 42]. The overall prevalence of MCI determined by the reference standard was 24%. The iAD8 showed 77% sensitivity (95% CI: 69, 83, I^2^: 81.1%), 84% specificity (95% CI: 80, 87, I^2^: 77.5%), and the AUC was 0.92 (Table 4). The iAD8 was used in three studies (1,094 patients) to detect dementia [38, 41, 43]. The overall prevalence of dementia determined by the reference standard was 23%. The iAD8 showed 87% sensitivity (95% CI: 82, 91, I^2^: 98.0%), 64% specificity (95% CI: 61, 68, I^2^: 82.4%), and the AUC was 0.73 (Table 4). The iAD8 was used in five studies (884 patients) to detect CI [14, 36, 37, 39, 42]. The overall prevalence of CI determined by the reference standard was 82%. The iAD8 showed 90% sensitivity (95% CI: 87, 92, I^2^: 79.8%), 62% specificity (95% CI: 54, 69, I^2^: 92.0%), and the AUC was 0.92 (Table 4).

The pAD8 was used in two studies (637 patients) at a cut-off of two to detect MCI [37, 40]. The overall prevalence of MCI determined by the reference standard was 26%. The pAD8 showed 64% sensitivity (95% CI: 56, 71, I^2^: 54.6%) and 58% specificity (95% CI: 54, 63, I^2^: 86.2%) (Table 4). The pAD8 was used in two studies (433 patients) to detect CI [36, 37]. The overall prevalence of CI determined by the reference standard was 82%. The pAD8 showed 58% sensitivity (95% CI: 53, 63, I^2^: 0.0%) and 64% specificity (95% CI: 52, 74, I^2^: 0.0%) (Table 4).

### 3.12 Pooled predictive parameters of iAD8 in the tertiary care/hospitalized setting

The iAD8 was used in two studies (177 patients) at a cut-off of two to detect dementia [46, 48]. The overall prevalence of dementia determined by the reference standard was 28%. The iAD8 showed 96% sensitivity (95% CI: 86, 100, I^2^: 0.0%) and 45% specificity (95% CI: 36, 54, I^2^: 95.1%) (Table 4). The iAD8 was used in two studies (177 patients) to detect CI [46, 48]. The overall prevalence of CI determined by the reference standard was 64%. The iAD8 showed 89% sensitivity (95% CI: 82, 94, I^2^: 0.0%) and 65% specificity (95% CI: 52, 77, I^2^: 65.1%) (Table 4).

### 3.13 Meta-regression and sensitivity analysis

The results of the meta-analysis consistently revealed an I^2^ value greater than 50% for most of the calculated pooled predictive parameters in all CI subgroups. This represents substantial inter-study heterogeneity. We conducted a meta-regression to identify sources of this heterogeneity, presented in the supplementary material. Meta-regression and sensitivity analysis changed the combined estimates moderately but did not alter the total inference. Leave one-study-out meta-analysis showed no significant effect on the results by any individual study.

### 3.14 Risk of bias and applicability

Quality assessment of the included studies is presented in the supplementary material. A high or unclear risk of bias in at least one domain was found in all included studies. We observed a recurring unclear risk of bias in the “Index test” and “Reference Standard” domains as many included articles did not specify whether the personnel conducting AD8 was blinded towards the reference standard results, or vice versa. The inter-rater agreement on the overall risk of bias assessment was 79.1% (296/374 items) with a Cohen’s Kappa of 0.58 (95% CI: 0.49, 0.66).

## 4. Discussion

In this systematic review and meta-analysis, we evaluated the predictive performance of iAD8 and pAD8 to screen for CI across all healthcare settings. The included studies were conducted in 1) community/primary care [7, 10, 18–34], 2) secondary care/memory clinics [14, 35–45], and 3) tertiary care/hospitals [46–48]. We found the diagnostic accuracy of the iAD8 to be superior to the pAD8 in screening for MCI, dementia, and CI (MCI or dementia) when compared against a reference standard. When screening for MCI, the iAD8 yielded a pooled 80% sensitivity and 79% specificity versus the pAD8 pooled 57% sensitivity and 71% specificity. For dementia, the iAD8 yielded a pooled 91% sensitivity and 64% specificity versus the pAD8 pooled 82% sensitivity and 75% specificity. For CI (MCI or dementia), the iAD8 yielded a pooled 88% sensitivity and 75% specificity versus the pAD8 pooled 62% sensitivity and 72% specificity. The AUC of the summary receiver operating characteristic analysis, which reflects the overall diagnostic accuracy of a screening tool, was superior for the iAD8 than the pAD8 in all CI groups at a cut-off of two. The AUC of iAD8 was highest at 0.91 to detect of CI (MCI or dementia) versus normal cognition.

Informants are often close family members who may reliably recognize subtle cognitive and/or functional deficits, or changes in the premorbid abilities of patients. Given that individuals with severe CI may be unaware of the degree of their impairments, one may presume the pAD8 is best suited to detect MCI than more severe CI [49–51]. On the contrary, we found a poorer performance of pAD8 when screening for MCI versus dementia. This discrepancy may be due to the patients diagnosed with MCI having already progressed to early dementia and lost insight. The superior performance of the pAD8 in detecting dementia may be due to adopting a higher cut-off of two in our meta-analysis. This was done to allow for direct comparison between the performance of iAD8 and pAD8, instead of using a pAD8 cut-off of one which was suggested by the creators of the tool to provide the best combination of sensitivity and specificity [10]. Individual studies that used multiple cut-offs found pAD8 to be most sensitive at a cut-off of 1, at the sacrifice of specificity. This contextualizes why patients with a greater degree of CI (i.e. dementia) were more likely to test positive at a higher minimum cut-off value in our analysis. These results, however, do not negate the utility of the pAD8 as a screening tool for MCI. Informants may not be available to attend medical appointments, and some patients may not have reliable informants. In these circumstances, using pAD8 at a cut-off of one may be a reasonable alternative.

Both the iAD8 and pAD8 had superior NPV than PPV when screening for MCI and dementia. This may result in more false positives, but fewer false negatives. Achieving this balance is preferred for a screening tool since positive results will identify at-risk patients who warrant further follow-up, diagnostic testing, and prompt intervention. This can help minimize the harms associated with undetected disease. To date, there is insufficient evidence for the asymptomatic screening of all older adults, as it can lead to misdiagnosis and unnecessary healthcare utilization [52]. Thus, it is important that clinicians remain aware of the risk factors, warning signs, and symptoms of cognitive impairment to apply AD8 in the appropriate clinical context.

We also conducted a separate analysis based on setting. Despite variances in the calculated sensitivity and specificity, we were not able to appreciate a clear impact on the performance of the iAD8 and pAD8 based on setting. For example, when screening for dementia using iAD8 in the community/primary care setting, the pooled sensitivity and specificity was 92% and 67% respectively, versus 87% and 64% in the secondary care/memory clinic setting, and 96% and 45% in the tertiary care/hospitalized setting. Similarly, for the pAD8 when screening for MCI in the community/primary care setting, the pooled sensitivity and specificity was 51% and 74% respectively, versus 64% and 58% in the secondary care/memory clinic setting. Factors that may explain these slight differences in the pooled test parameters include the number of patients and studies in each setting, degree of study heterogeneity, and calculated prevalence of the condition which is known to impact the predictive performance of screening tests [53]. Based on our calculations, there was no consistent increase in the prevalence of cognitive impairment in the secondary care/memory clinic and tertiary care/hospitalized settings that one could expect compared to the community/primary care setting.

An ideal CI screening tool should be: 1) quick to administer (≤ 5 minutes), 2) acceptable performance against gold-standard testing (i.e. predictive parameters), 3) unaffected by an individual’s language, culture, and/or education, 4) easily scored, 5) free and open-access, and 6) completed virtually/over the phone. Currently, the Montreal Cognitive Assessment (MoCA) and Mini Mental Status Examination (MMSE) are the two most widely used cognitive impairment screening tools [1]. Both have become less accessible due to copyright restrictions requiring payment for training or use [54]. Notably, both require ≥10 minutes to complete, which makes them less suitable for fast-paced clinical environments such as emergency departments and preoperative settings where detection of pre-existing CI is crucial to facilitate management [55, 56].

Conversely, the AD8 is a free and validated screening tool that takes less than three minutes to administer. It has good inter-individual reliability, substantial intra-individual and inter-modal reliability, good internal consistency, and strong concurrent and construct validity in both research and clinical settings irrespective of patient or informant age, gender, education, race, and informant relationship [7, 10, 14]. Patients serve as their own controls as scoring is not contingent on published thresholds for impairment, differentiating it from performance-based screening tests such as the MMSE or MoCA [25]. Using AD8 clinicians can assess change from previous levels of functioning without the need to compare baseline assessments to current performance. This may allow for earlier CI detection, particularly in high-functioning individuals at early-stage disease who may score within normal ranges on performance-based screening [25].

The flexibility of the AD8 to be informant or self-administered is a significant advantage compared to other performance-based cognitive screening tools. Completion of the iAD8 is likely to identify CI. However, pAD8 can also be completed to calculate a discrepancy score, which can ascertain a patient’s level of insight and degree of cognitive decline [57]. Limitations of the pAD8 include the possible impact of coexisting mood disorders such as depression, emotional factors such as anxiety, and underlying medical illnesses.

Based on our inclusion criteria, we found no studies conducted in the preoperative or emergency department settings where AD8 could be clinically useful. A high prevalence of unrecognized CI exists in older surgical patients, and there is a need for rigorous screening to risk stratify these patients [58]. Pre-existing CI increases the risk of postoperative complications such as postoperative cognitive dysfunction and/or delirium [59]. Although providing comprehensive preoperative geriatric evaluation can improve the 90-day survival of older adults undergoing major surgery, it leads to increased cost of care and higher readmission rates [60]. Moreover, geriatric consultation and associated CI work-up may not be possible in the time-restrained preoperative setting. AD8 can be easily included in the preoperative evaluation for all older surgical patients, given either to informants or patients themselves. Further work is needed to validate its use in the preoperative setting and to determine if AD8 scores can predict important adverse outcomes such as delirium, length of stay, frailty, and mortality.

### 4.1 Limitations

Some limitations to our systematic review and meta-analysis exist. The inclusion of retrospective cohort and case-control studies is inherently prone to bias. Moreover, there was considerable heterogeneity in the included studies. Particularly, the reference standards varied across studies which may influence the calculated prevalence of CI and indirectly impact the diagnostic performance of AD8. Other contributory factors such as geographical variation, diverse clinical populations, and different cut-offs limited the number of studies included in our meta-analysis. Our review was limited to studies that used recognized clinical reference standards, which prevented the inclusion of diagnostic studies that used other screening tools as reference standards. Lastly, fewer studies evaluated the pAD8 and only three studies were from the tertiary care/hospitalized setting which limited the amount of data available.

## 5. Conclusion

AD8 is a free, quick, and easy-to-score CI screening tool that requires minimal training and can be administered to both informants and patients directly. It can capture a patient’s change in cognition over time, which is fundamental to diagnosing MCI and dementia. AD8 can also be delivered over the phone or through other virtual formats. The diagnostic accuracy of the iAD8 was found to be superior to that of the pAD8 when screening for MCI, dementia, and CI (MCI or dementia). In summary, AD8 is a CI screening tool that can be used in many different settings particularly those that are time and resource limited.

## Supporting information

S1 Checklist (DOCX)

S1 File Contains all the supporting tables and figures. (PDF)
