# Supplementary material for: Diagnostic accuracy of Ascertain Dementia 8-item Questionnaire by participant and informant–A systematic review and meta-analysis
Source: PLoS One. 2023 Sep 12;18(9):e0291291. doi: 10.1371/journal.pone.0291291 (PMC10497164; doi:10.1371/journal.pone.0291291)
Supplement: S1 File — (PDF) [file pone.0291291.s002.pdf]

Supplement: Diagnostic accuracy of Ascertain Dementia 8-item Questionnaire by participant and informant – A systematic review and meta-analysis

Table of Contents

S1 Fig. Pooled forest plots of sensitivity and specificity of informant and participant completed AD8 in community/primary care setting (cut off 2 or greater). 3

S2 Fig. Summary receiver operating characteristic of the iAD8 and pAD8 in community/primary care setting.. 4

S3 Fig. Pooled forest plots of sensitivity and specificity of informant and participant completed AD8 in secondary care/memory clinic setting (cut off 2 or greater). 5

S4 Fig. Summary receiver operating characteristic of the iAD8 and pAD8 in secondary care/memory clinic setting.. 6

S5 Fig. Pooled forest plots of sensitivity and specificity of informant completed AD8 in tertiary care/hospitalized setting (cut off 2 or greater). 7

S6 Fig. Risk of bias and applicability graphs for QUADAS-2 8

S1 Table. Meta-regression analysis 9

S2 Table. QUADAS-2: Quality Assessment of Diagnostic Accuracy Studies 11

S3 Table. Predictive parameters of AD8 to screen for mild cognitive impairment 12

S4 Table. Predictive parameters of AD8 to screen for dementia 13

S5 Table. Predictive parameters of AD8 to screen for CI (MCI or Dementia) 15

S6 Table. Test characteristics of the various AD8 score cut-offs for informants and participants 17

S7 Table. Search Strategy 18

This supplemental material has been provided by the authors to give readers additional information about their work.

AD8 informant vs. reference standard for MCI

Study	TP	FP	FN	TN	Sensitivity (95% CI)	Specificity (95% CI)
Correia, 2011	13	35	5	56	0.72 [0.47, 0.90]	0.62 [0.51, 0.72]
Galvin, 2005	50	24	18	144	0.74 [0.61, 0.83]	0.86 [0.79, 0.91]
Malmstrom, 2009	16	8	6	31	0.73 [0.50, 0.89]	0.79 [0.64, 0.91]
Yang, 2011	70	36	3	130	0.96 [0.88, 0.99]	0.78 [0.71, 0.84]

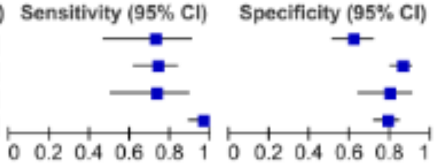

Pooled Sensitivity = 0.82 (0.76–0.88); Chi-squared = 18.20; df = 3 (p=0.0004); Inconsistency (I-square) = 83.5%
Pooled Specificity = 0.78 (0.74–0.82); Chi-squared = 19.04; df = 3 (p=0.0003); Inconsistency (I-square) = 84.2%

AD8 informant vs. reference standard for dementia

Study	TP	FP	FN	TN	Sensitivity (95% CI)	Specificity (95% CI)
Dominguez, 2021	147	123	6	90	0.96 [0.92, 0.99]	0.42 [0.36, 0.49]
Galvin, 2010	75	30	3	149	0.96 [0.89, 0.99]	0.83 [0.77, 0.88]
Kim, 2019	153	47	17	203	0.90 [0.84, 0.94]	0.81 [0.76, 0.86]
Morris, 2020	125	44	18	43	0.87 [0.81, 0.92]	0.49 [0.39, 0.60]

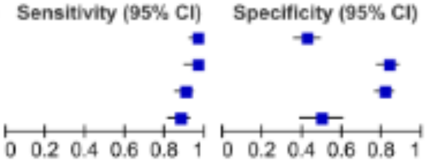

Pooled Sensitivity = 0.92 (0.89–0.94); Chi-squared = 10.81; df = 3 (p=0.0128); Inconsistency (I-square) = 72.3%
Pooled Specificity = 0.67 (0.63–0.70); Chi-squared = 115.18; df = 3 (p=0.0000); Inconsistency (I-square) = 97.4%

AD8 informant vs. reference standard for CI

Study	TP	FP	FN	TN	Sensitivity (95% CI)	Specificity (95% CI)
Galvin, 2005	105	16	19	96	0.85 [0.77, 0.91]	0.86 [0.78, 0.92]
Galvin, 2007	148	10	28	139	0.84 [0.78, 0.89]	0.93 [0.88, 0.97]
Shang, 2021	46	38	20	56	0.70 [0.57, 0.80]	0.60 [0.49, 0.70]
Yang, 2011	122	25	3	89	0.98 [0.93, 1.00]	0.78 [0.69, 0.85]

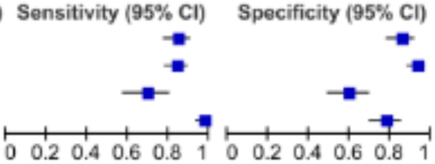

Pooled Sensitivity = 0.86 (0.82–0.89); Chi-squared = 32.50; df = 3 (p=0.0000); Inconsistency (I-square) = 90.8%
Pooled Specificity = 0.81 (0.77–0.84); Chi-squared =43.78; df = 3 (p=0.0000); Inconsistency (I-square) = 93.1%

AD8 participant vs. reference standard for MCI

Study	TP	FP	FN	TN	Sensitivity (95% CI)	Specificity (95% CI)
Cai, 2021	78	102	82	261	0.49 [0.41, 0.57]	0.72 [0.67, 0.76]
Chin, 2013	18	33	9	118	0.67 [0.46, 0.83]	0.78 [0.71, 0.84]

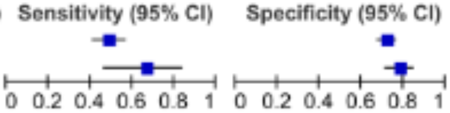

Pooled Sensitivity = 0.51 (0.44–0.59); Chi-squared = 3.02; df = 1 (p=0.0820); Inconsistency (I-square) = 66.9%
Pooled Specificity = 0.74 (0.70–0.77); Chi-squared = 2.20; df = 1 (p=0.1381); Inconsistency (I-square) = 54.5%

AD8 participant vs. reference standard for dementia

Study	TP	FP	FN	TN	Sensitivity (95% CI)	Specificity (95% CI)
Cai, 2021	17	142	0	364	1.00 [0.80, 1.00]	0.72 [0.68, 0.76]
Kim, 2019	121	63	49	188	0.71 [0.64, 0.78]	0.75 [0.69, 0.80]
Yang, 2016	398	339	46	1232	0.90 [0.86, 0.92]	0.78 [0.76, 0.80]

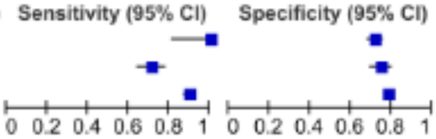

Pooled Sensitivity = 0.85 (0.82–0.88); Chi-squared = 34.84; df = 2 (p=0.0000); Inconsistency (I-square) = 94.3%
Pooled Specificity = 0.77 (0.75–0.78); Chi-squared = 9.24; df = 2 (p=0.0098); Inconsistency (I-square) = 78.4%

AD8 participant vs. reference standard for CI

Study	TP	FP	FN	TN	Sensitivity (95% CI)	Specificity (95% CI)
Chin, 2013	77	15	28	58	0.73 [0.64, 0.81]	0.79 [0.68, 0.88]
Galvin, 2007	109	40	67	109	0.62 [0.54, 0.69]	0.73 [0.65, 0.80]

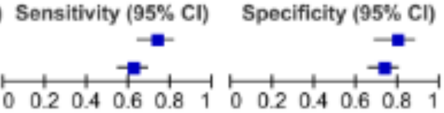

Pooled Sensitivity = 0.66 (0.60–0.72); Chi-squared = 3.89; df = 1 (p=0.0485); Inconsistency (I-square) = 74.3%
Pooled Specificity = 0.75 (0.69–0.81); Chi-squared =1.07; df = 1 (p=0.3018); Inconsistency (I-square) = 6.2%

S1 Fig. Pooled forest plots of sensitivity and specificity of informant and participant completed AD8 in community/primary care setting (cut off 2 or greater). TP, true positive; FP, false positive; FN, false negative; TN, true negative

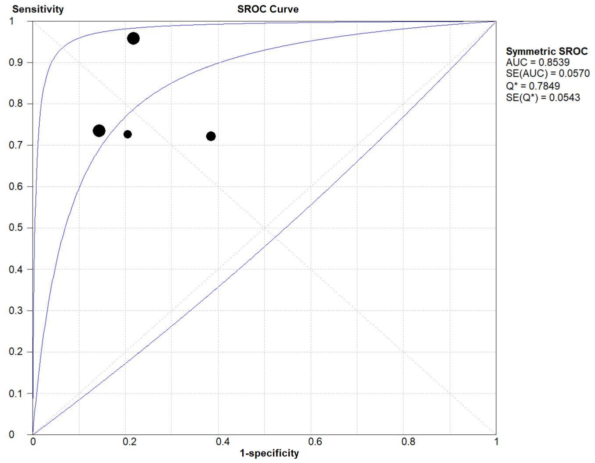

iAD8 MCI community/primary care

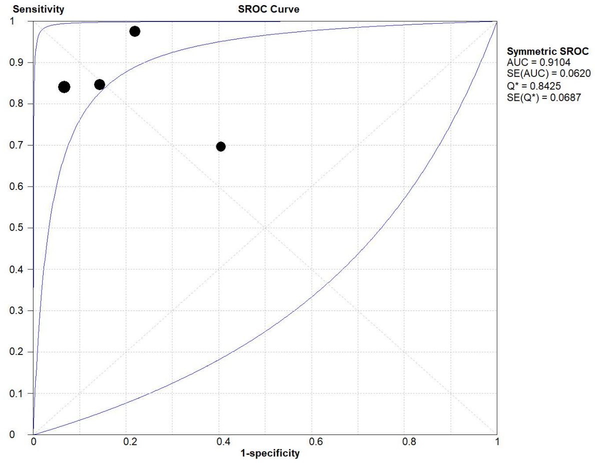

iAD8 CI (MCI or Dem) community/primary care

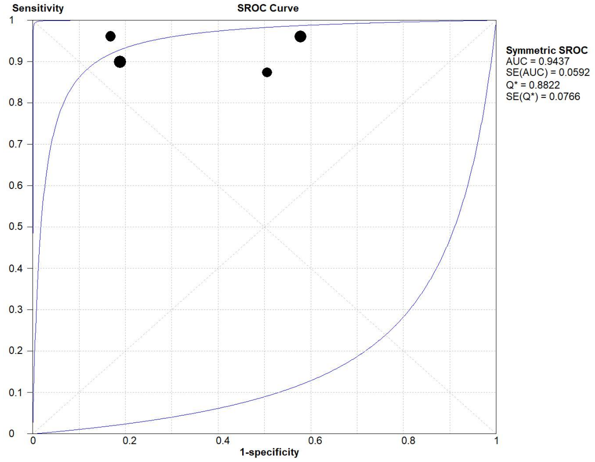

iAD8 Dem community/primary care

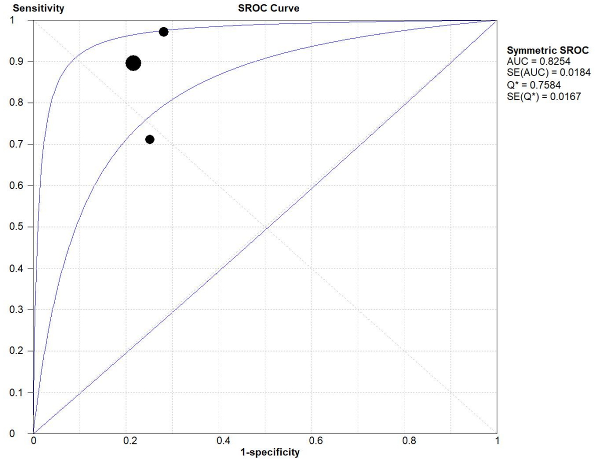

pAD8 Dem community/primary care

S2 Fig. Summary receiver operating characteristic of the iAD8 and pAD8 in community/primary care setting. Legend: AUC, area under the curve; Dem, Dementia; MCI, mild cognitive impairment; Q*, point of indifference on the ROC curve; SE, standard error; SROC, summary receiver operating characteristic.

AD8 informant vs. reference standard for MCI

Study	TP	FP	FN	TN	Sensitivity (95% CI)	Specificity (95% CI)
Dong, 2013	53	29	12	186	0.82 [0.70, 0.90]	0.87 [0.81, 0.91]
Razavi, 2014	13	39	0	134	1.00 [0.75, 1.00]	0.77 [0.70, 0.83]
Ryu, 2009	49	8	23	75	0.68 [0.56, 0.79]	0.90 [0.82, 0.96]

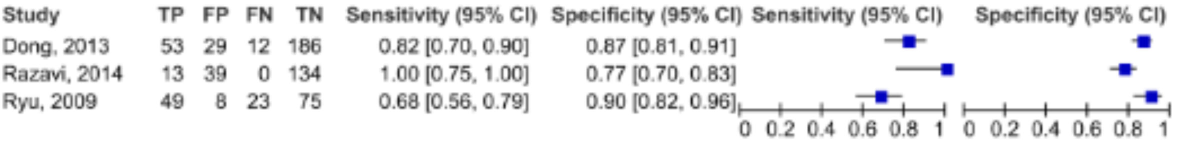

Pooled Sensitivity = 0.77 (0.69–0.83); Chi-squared = 10.59; df = 2 (p=0.0050); Inconsistency (I-square) = 81.1%
Pooled Specificity = 0.84 (0.80–0.87); Chi-squared = 8.90; df = 2 (p=0.0117); Inconsistency (I-square) = 77.5%

AD8 informant vs. reference standard for dementia

Study	TP	FP	FN	TN	Sensitivity (95% CI)	Specificity (95% CI)
Karam, 2018	73	12	2	45	0.97 [0.91, 1.00]	0.79 [0.66, 0.89]
Razavi, 2014	128	13	1	44	0.99 [0.96, 1.00]	0.77 [0.64, 0.87]
Tak, 2021	16	275	29	456	0.36 [0.22, 0.51]	0.62 [0.59, 0.66]

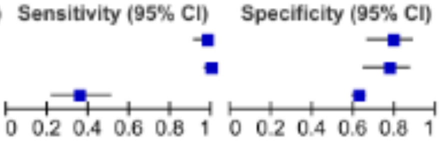

Pooled Sensitivity = 0.87 (0.82–0.91); Chi-squared = 102.28; df = 2 (p=0.0000); Inconsistency (I-square) = 98.0%
Pooled Specificity = 0.64 (0.61–0.68); Chi-squared = 11.38; df = 2 (p=0.0034); Inconsistency (I-square) = 82.4%

AD8 informant vs. reference standard for CI

Study	TP	FP	FN	TN	Sensitivity (95% CI)	Specificity (95% CI)
Chio, 2018	97	27	13	16	0.88 [0.81, 0.94]	0.37 [0.23, 0.53]
Dong, 2013	225	5	18	32	0.93 [0.89, 0.96]	0.86 [0.71, 0.95]
Galvin, 2006	208	16	18	13	0.92 [0.88, 0.95]	0.45 [0.26, 0.64]
Larner, 2017	34	9	1	2	0.97 [0.85, 1.00]	0.18 [0.02, 0.52]
Ryu, 2009	90	4	25	36	0.78 [0.70, 0.85]	0.90 [0.76, 0.97]

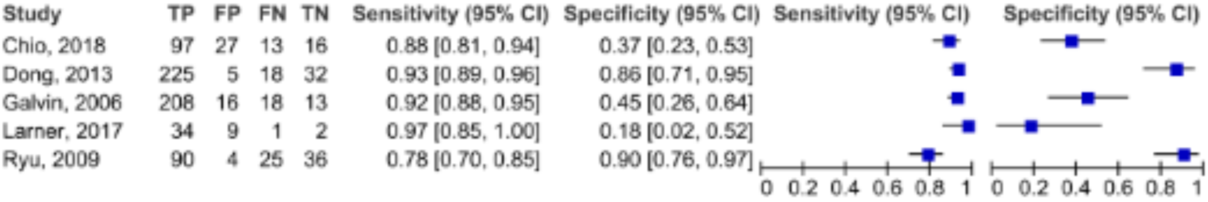

Pooled Sensitivity = 0.90 (0.87–0.92); Chi-squared = 19.76; df = 4 (p=0.0006); Inconsistency (I-square) = 79.8%
Pooled Specificity = 0.62 (0.54–0.69); Chi-squared = 50.29; df =4 (p=0.0000); Inconsistency (I-square) = 92.0%

AD8 participant vs. reference standard for MCI

Study	TP	FP	FN	TN	Sensitivity (95% CI)	Specificity (95% CI)
Dong, 2013	37	75	28	140	0.57 [0.44, 0.69]	0.65 [0.58, 0.71]
Mansbach, 2016	67	122	31	137	0.68 [0.58, 0.77]	0.53 [0.47, 0.59]

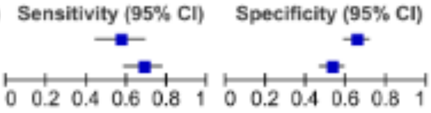

Pooled Sensitivity = 0.64 (0.56–0.71); Chi-squared = 2.20; df = 1 (p=0.1377); Inconsistency (I-square) = 54.6%
Pooled Specificity = 0.58 (0.54–0.63); Chi-squared = 7.26; df = 1 (p=0.0070); Inconsistency (I-square) = 86.2%

AD8 participant vs. reference standard for CI

Study	TP	FP	FN	TN	Sensitivity (95% CI)	Specificity (95% CI)
Chio, 2018	61	16	49	27	0.55 [0.46, 0.65]	0.63 [0.47, 0.77]
Dong, 2013	144	13	99	24	0.59 [0.53, 0.65]	0.65 [0.47, 0.80]

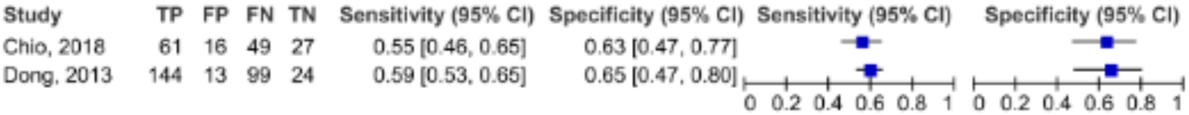

Pooled Sensitivity = 0.58 (0.53–0.63); Chi-squared = 0.45; df = 1 (p=0.5028); Inconsistency (I-square) = 0.0%
Pooled Specificity = 0.64 (0.52–0.74); Chi-squared = 0.04; df = 1 (p=0.8474); Inconsistency (I-square) = 0.0%

S3 Fig. Pooled forest plots of sensitivity and specificity of informant and participant completed AD8 in secondary care/memory clinic setting (cut off 2 or greater). TP, true positive; FP, false positive; FN, false negative; TN, true negative

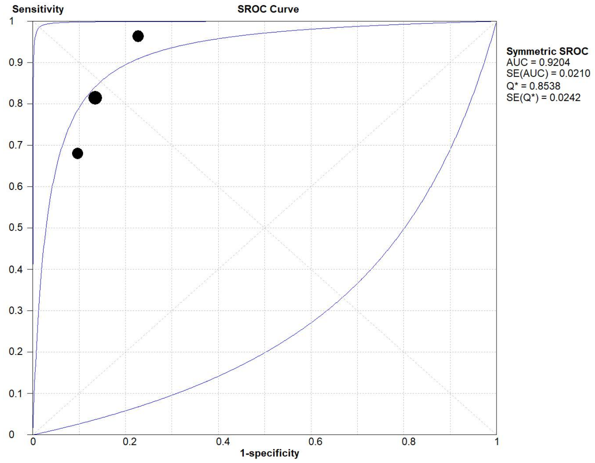

iAD8 MCI secondary/memory clinic

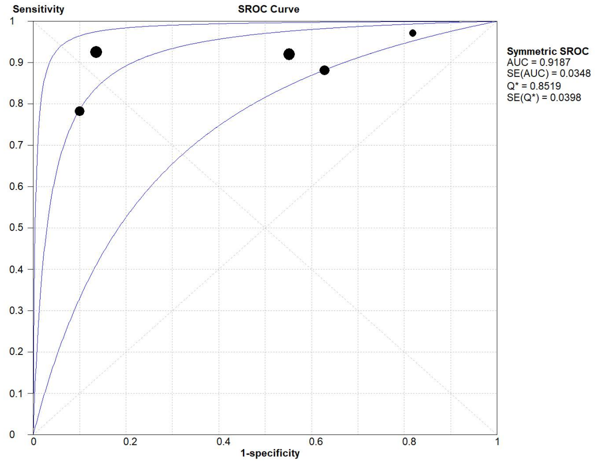

iAD8 CI (MCI or Dem) secondary/memory clinic

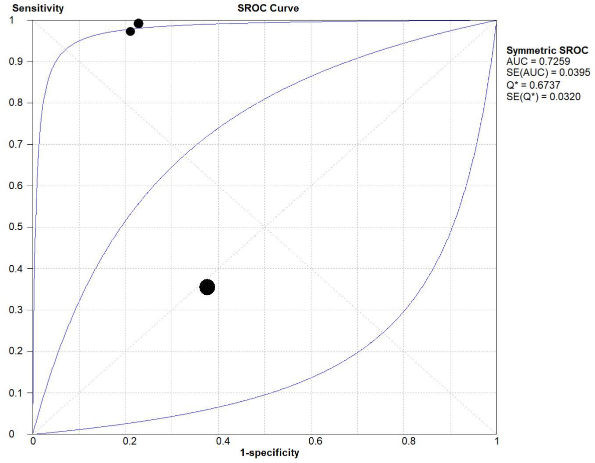

iAD8 Dem secondary/memory clinic

S4 Fig. Summary receiver operating characteristic of the iAD8 and pAD8 in secondary care/memory clinic setting. Legend: AUC, area under the curve; Dem, Dementia; MCI, mild cognitive impairment; Q*, point of indifference on the ROC curve; SE, standard error; SROC, summary receiver operating characteristic.

AD8 informant vs. reference standard for dementia

Study	TP	FP	FN	TN	Sensitivity (95% CI)	Specificity (95% CI)
Duggan, 2020	36	32	1	6	0.97 [0.86, 1.00]	0.16 [0.06, 0.31]
Taylor-Rowan, 2022	11	38	1	52	0.92 [0.62, 1.00]	0.58 [0.47, 0.68]

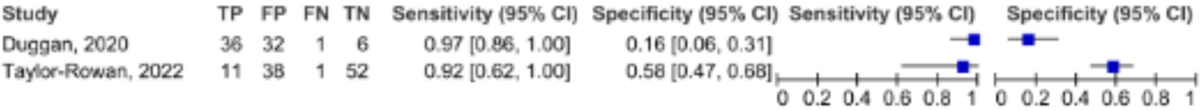

Pooled Sensitivity = 0.96 (0.86–1.00); Chi-squared = 0.63; df = 1 (p=0.4261); Inconsistency (I-square) = 0.0%
Pooled Specificity = 0.45 (0.36–0.54); Chi-squared =20.59; df = 1 (p=0.0000); Inconsistency (I-square) = 95.1%

AD8 informant vs. reference standard for CI

Study	TP	FP	FN	TN	Sensitivity (95% CI)	Specificity (95% CI)
Duggan, 2020	65	3	6	1	0.92 [0.83, 0.97]	0.25 [0.01, 0.81]
Taylor-Rowan, 2022	37	19	6	40	0.86 [0.72, 0.95]	0.68 [0.54, 0.79]

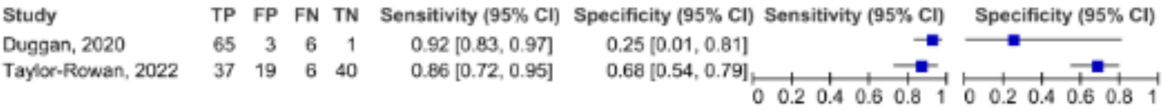

Pooled Sensitivity = 0.89 (0.82–0.94); Chi-squared = 0.84; df = 1 (p=0.3600); Inconsistency (I-square) = 0.0%
Pooled Specificity = 0.65 (0.52–0.77); Chi-squared = 2.87 df = 1 (p=0.0904); Inconsistency (I-square) = 65.1%

S5 Fig. Pooled forest plots of sensitivity and specificity of informant completed AD8 in tertiary care/hospitalized setting (cut off 2 or greater). TP, true positive; FP, false positive; FN, false negative; TN, true negative

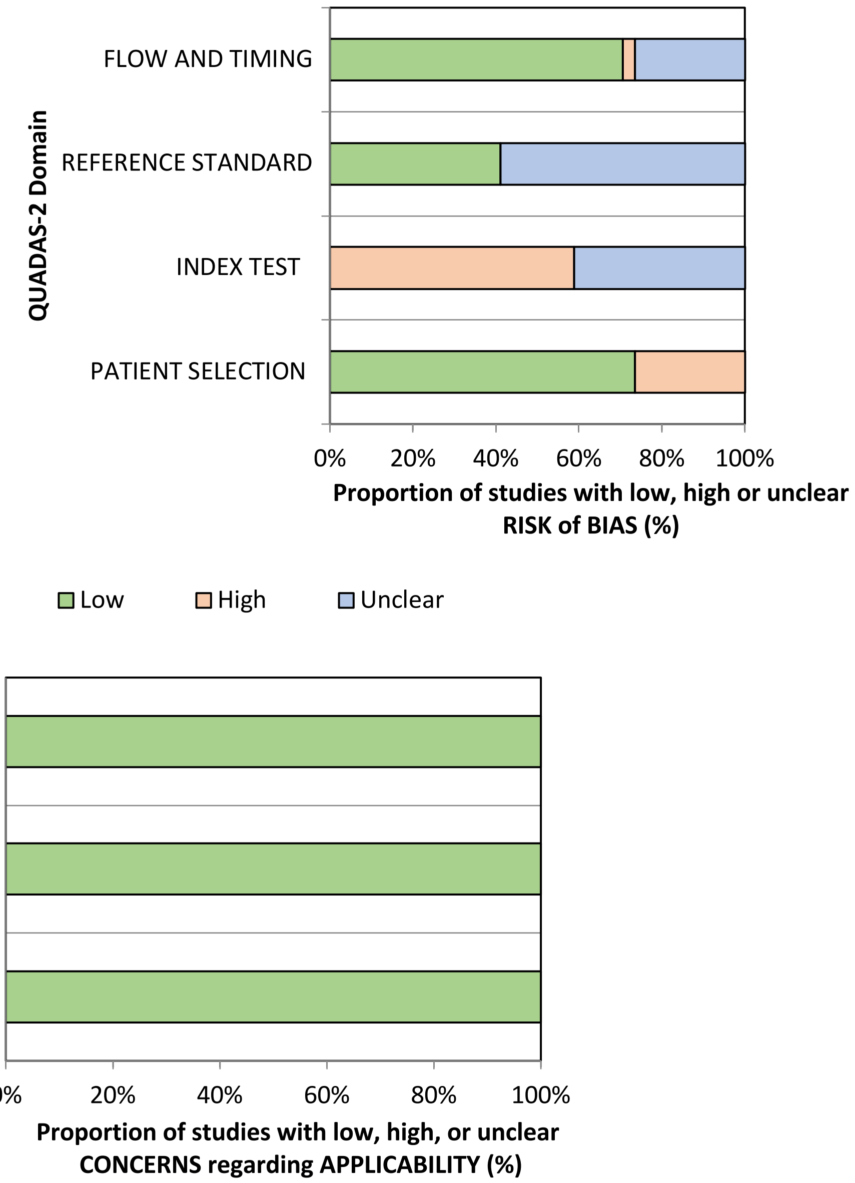

S6 Fig. Risk of bias and applicability graphs for QUADAS-2

S1 Table. Meta-regression analysis

Meta-regression and sensitivity analysis of **having Mild Cognitive Impairment (MCI)** with Informant completed AD8 cut-off ≥2

	Sensitivity			Diagnostic Odds Ratio (Log scale)			Specificity		
**Co-variate (Number of studies)**	**Point Estimate (95% CI)**	**Coefficient (SE)**	**p-value**	**Point Estimate (95% CI)**	**Coefficient (SE)**	**p-value**	**Point Estimate (95% CI)**	**Coefficient (SE)**	**p-value**
Age (6)	80.7 (69.5-88.5)	0.056 (0.054)	0.298	3.18 (2.6-3.8)	0.050 (0.046)	0.280	81.9 (77.4-85.7)	0.001 (0.026)	0.970
Female gender (7)	79.5 (69.4-86.9)	-0.031 (0.021)	0.145	2.94 (2.2-3.7)	-0.055 (0.025)	0.025	79.7 (71.5-86.0)	-0.035 (0.011)	0.002
Sample Size (7)	79.5 (69.4-86.9)	0.004 (0.003)	0.176	2.94 (2.2-3.7)	0.008 (0.003)	0.023	79.7 (71.5-86.0)	0.004 (0.002)	0.113

Meta-regression and sensitivity analysis of **having Dementia** with Informant AD8 cut-off ≥2

	Sensitivity			Diagnostic Odds Ratio (Log scale)			Specificity		
**Co-variate (Number of studies)**	**Point Estimate (95% CI)**	**Coefficient (SE)**	**p-value**	**Point Estimate (95% CI)**	**Coefficient (SE)**	**p-value**	**Point Estimate (95% CI)**	**Coefficient (SE)**	**p-value**
Age (8)	94.6 (90.6-96.9)	-0.035 (0.062)	0.571	3.6 (2.7-4.6)	0.076 (0.099)	0.444	66.4 (50.5-79.3)	0.080 (0.060)	0.186
Female gender (9)	92.9 (82.4-97.4)	0.094 (0.010)	< 0.001	3.2(1.9-4.5)	0.099 (0.028)	<0.001	66.3 (54.9-76.0)	0.009 (0.027)	0.724
Sample Size (9)	92.9 (82.4-97.4)	-0.006 (0.001)	<0.001	3.2(1.9-4.5)	0.097 (0.028)	<0.001	66.3 (54.9-76.0)	0.006 (0.018)	0.750

Meta-regression and sensitivity analysis of **having CI (MCI or Dementia)** with Informant AD8 cut-off ≥2

	Sensitivity			Diagnostic Odds Ratio (Log scale)			Specificity		
**Co-variate (Number of studies)**	**Point Estimate (95% CI)**	**Coefficient (SE)**	**p-value**	**Point Estimate (95% CI)**	**Coefficient (SE)**	**p-value**	**Point Estimate (95% CI)**	**Coefficient (SE)**	**p-value**
Age (11)	88.2 (83.2-91.8)	-0.057 (0.039)	0.138	2.9 (2.1-3.7)	0.023 (0.078)	0.767	69.6 (54.7-81.3)	0.100 (0.066)	0.131
Female gender (11)	88.2 (83.2-91.8)	0.013 (0.010)	0.190	2.9 (2.1-3.7)	0.029 (0.019)	0.119	69.6 (54.7-81.3)	0.015 (0.019)	0.427
Sample Size (11)	88.2 (83.2-91.8)	0.001 (0.003)	0.819	2.9 (2.1-3.7)	0.011 (0.004)	0.003	69.6 (54.7-81.3)	0.011 (0.003)	0.002

Meta-regression and sensitivity analysis of **having CI (MCI or Dementia)** with Participant AD8 cut-off ≥2

	**Sensitivity**			**Diagnostic Odds Ratio (Log scale)**			**Specificity**		
**Co-variate (Number of studies)**	**Point Estimate (95% CI)**	**Coefficient (SE)**	**p-value**	**Point Estimate (95% CI)**	**Coefficient (SE)**	**p-value**	**Point Estimate (95% CI)**	**Coefficient (SE)**	**p-value**
Age (4)	62.0(58-65)	-0.057 (0.024)	0.019	4.06 (2.15-7.68)	-0.109 (0.048)	0.023	72.0(67-77)	-0.044 (0.033)	0.181
Female gender (4)	62.0(58-65)	0.019 (0.020)	0.333	4.06 (2.15-7.68)	0.043 (0.051)	0.400	72.0(67-77)	0.015 (0.027)	0.565
Sample Size (4)	62.0(58-65)	-0.000 (0.002)	0.776	4.06 (2.15-7.68)	-0.000 (0.004)	0.911	72.0(67-77)	0.000 (0.002)	0.848

S2 Table. QUADAS-2: Quality Assessment of Diagnostic Accuracy Studies

Study ID	Patient selection		Index test		Reference standard		Flow and timing
	Risk of bias	Applicability	Risk of bias	Applicability	Risk of bias	Applicability	Risk of bias
Cai, 2021	Low	Low	UNC	Low	UNC	Low	Low
Carnero Pardo, 2013	Low	Low	High	Low	UNC	Low	Low
Chan, 2016	Low	Low	UNC	Low	UNC	Low	Low
Chen, 2018	Low	Low	UNC	Low	UNC	Low	UNC
Chin, 2013	Low	Low	High	Low	UNC	Low	Low
Chio, 2018	Low	Low	High	Low	UNC	Low	UNC
Correia, 2011	Low	Low	UNC	Low	Low	Low	Low
Dominguez, 2021	Low	Low	High	Low	Low	Low	Low
Dong, 2013	High	Low	High	Low	UNC	Low	Low
Dong, 2021	Low	Low	High	Low	UNC	Low	Low
Duggan, 2020	Low	Low	UNC	Low	Low	Low	Low
Galvin, 2010	Low	Low	UNC	Low	Low	Low	Low
Galvin, 2007	Low	Low	High	Low	Low	Low	Low
Galvin, 2005	Low	Low	High	Low	Low	Low	Low
Galvin, 2006	Low	Low	High	Low	Low	Low	Low
Jackson, 2016	Low	Low	High	Low	Low	Low	High
Kan, 2019	Low	Low	High	Low	UNC	Low	Low
Karam, 2018	Low	Low	High	Low	Low	Low	Low
Kasai, 2021	Low	Low	UNC	Low	Low	Low	Low
Kim, 2019	High	Low	High	Low	UNC	Low	Low
Larner, 2017	Low	Low	UNC	Low	UNC	Low	UNC
Malmstrom, 2009	Low	Low	High	Low	Low	Low	UNC
Mansbach, 2016	Low	Low	UNC	Low	Low	Low	Low
Mao, 2018	Low	Low	High	Low	UNC	Low	UNC
Morris, 2020	Low	Low	UNC	Low	UNC	Low	Low
Razavi, 2014	High	Low	UNC	Low	Low	Low	Low
Ryu, 2009	High	Low	High	Low	Low	Low	Low
Shang, 2021	Low	Low	UNC	Low	UNC	Low	UNC
Tak, 2021	High	Low	High	Low	UNC	Low	UNC
Taylor-Rowan, 2022	Low	Low	UNC	Low	UNC	Low	UNC
Tew, 2015	High	Low	High	Low	UNC	Low	UNC
Usarel, 2019	High	Low	High	Low	UNC	Low	Low
Yang, 2011	High	Low	High	Low	UNC	Low	Low
Yang, 2016	High	Low	UNC	Low	UNC	Low	Low

Risk of Bias: low (if quality domain was met, indicating low risk of bias); high (if quality was not met, indicating high risk of bias); unclear (if information to judge the potential of bias was incomplete or unclear, indicating an uncertain risk of bias). Applicability high/low/unclear (are there concerns that the included patients, the index test, its conduct of interpretation or the target condition as defined by the reference standard do not match the review question).

S3 Table. Predictive parameters of AD8 to screen for mild cognitive impairment

Author, year	Sample size (N)	Prevalence of cognitive disorder – n (%)	Cut-off used	Sensitivity % (95% CI)	Specificity % (95% CI)	AUC (95% CI)
			Informant AD8			
Community/primary-care setting						
**Correia, 2011**	109	MCI: 18 (18.9)†	2	72.2	61.1	0.769 (0.660-0.870)
			3	83.1	44.4	
**Galvin, 2005**	236	MCI: 68 (28.8) †	2	74	86	0.834
**Kan, 2019**	761	MCI: 193 (25.4) †	1	62	73	0.69 (0.64-0.73)
**Malmstrom, 2009**	61	MCI: 22 (36.1) †	1	86	59	0.847 (0.73-0.96)
			2	73	79	
**Yang, 2011**	239	MCI: 73 (30.5) †	2	95.9	78.1	0.948
Secondary care/memory clinic setting						
**Dong, 2013**	280	MCI: 65 (23.2) †	2	81.5	86.5	0.89 (0.83-0.95)
**Galvin, 2006**	255	MCI: 116 (46.4) †	2			0.847 (0.78-0.92)
**Razavi, 2014**	186	MCI: 13 (7.0)	2	100 (77.2-100)	77.3 (63.0-87.2)	0.899 (0.821-0.978)
**Ryu, 2009**	155	MCI: 72 (46.5) †	1	81	65	0.82 (0.75-0.90)
			2	68	90	
			3	53	95	
**Usarel, 2019**	334	MCI: 60 (18.0)	3	81.67	93.59	0.939 (0.905-0.972)
			Participant AD8			
Community/primary-care setting						
**Cai, 2021**	523	MCI: 160 (30.6)	1	65.6	41.6	0.607 (0.551-0.663)
			2	48.8	72.0	
			3	31.3	89.0	
**Chin, 2013**	178	MCI: 27 (15.2)	1	84	65	0.76 (0.67-0.85)
			2	67	78	
			3	55	83	
Secondary care/memory clinic setting						
**Dong, 2013**	280	MCI: 65 (23.2) †	2	56.9	64.9	0.63 (0.53-0.74)
**Mansbach, 2016**	357	MCI: 98 (27.5)	1	78 (68-85)	30 (15-50)	0.59 (0.48-0.71)
			2	68 (58-77)	53 (35-71)	
			3	47 (37-57)	63 (44-79)	

†NC: no cognitive impairment (determined by CDR score of 0), MCI: mild cognitive impairment (determined by CDR score of 0.5)

Abbreviations: AUC, Area under ROC curve; CDR, Clinical Dementia Rating; MCI, Mild Cognitive Impairment.

The cut-offs used for each index test (informant and participant AD8) to indicate a positive dementia screen are decided by the individual study authors. The creators of the informant AD8 screening tool originally utilize a cut-off of 2 and a cut-off of 1 when using the informant and participant AD8 respectively to indicate a positive dementia screen, as they provided the optimal predictive performance when compared to reference standard testing in those study populations. Cut-off values are greater than or equal to the numbers listed in the tables.

CDR stands for the Clinical Dementia Rating scale which is used to determine the presence and absence of cognitive impairment and stage its severity. A global score of CDR 0 indicates normal cognition/no dementia, CDR 0.5 indicates mild cognitive impairment (MCI), CDR ≥1 indicates dementia.

S4 Table. Predictive parameters of AD8 to screen for dementia

Author, year	Sample size (N)	Prevalence of cognitive disorder – n (%)	Cut-off used	Sensitivity % (95% CI)	Specificity % (95% CI)	AUC (95% CI)
			Informant AD8			
Community/primary-care setting						
**Chan, 2016**	309	Dem: 44 (14.2) †	3/4	91	91	0.97 (0.95-0.99)
**Chen, 2018**	40	Dem: 20 (50)	1/2	50	80	0.684 (0.518-0.821)
**Correia, 2011**	109	Dem: 15 (13.8)	3	100 (74.7-100)	67.0 (56.5-76.2)	
**Dominguez, 2021**	366	Dem: 153 (41.8)	2	96.1 (91-98)	42.1 (34-50)	0.94 (0.92-0.96)
			3	91.5 (85-95)	77.9 (72-83)	
			4	83.0 (76-88)	90.6 (86-94)	
**Galvin, 2010**	257	Dem: 78 (30.3) †	2	96.5 (92-99)	83.4 (77-89)	
**Kan, 2019**	761	Dem: 42 (5.5) †	3	76	94	0.89 (0.83-0.96)
**Kim, 2019**	420	Dem: 170 (40.5)	2	90 (84-94)	81 (74-86)	0.93 (0.90-0.95)
			3	83 (76-88)	89 (83-93)	
**Mao, 2018**	10360<br><br>DTA: 8805	Dem: 917 (8.9)<br><br>DTA:<br>Dem: 584 (6.6)	1/2	88	84	0.905 (0.894-0.917)
**Morris, 2020**	230	AD: 143 (62.2)	2	87.4	49.4	
Secondary care/memory clinic setting						
**Karam, 2018**	132	Dem: 75 (56.8)	2	97.33	78.95	0.98 (0.96-1.00)
			3	96.00	96.49	
			4	94.67	98.25	
**Razavi, 2014**	186	Dem: 129 (69.3)	2	99.2 (95.7-99.8)	77.3 (63.0-87.2)	0.953 (0.915-0.992)
**Tak, 2021**	776	Dem: 45 (5.8)	1	26.67	53.76	0.61 (0.55-0.68)
			2	35.56	62.38	
			3	48.89	67.58	
			4	100	99.86	
**Tew, 2015**	245	Dem: 159 (64.9)	1/2	89.9	80.2	0.92 (0.89-0.95)
			5/6	90.7	84.3	
**Usarel, 2019**	334	Dem: 118 (35.3)	5	100	96.3	0.999 (0.998-1.000)
Tertiary care/hospitalized setting						
**Duggan, 2020**	75	Dem: 37 (49.3) †	2	97.30 (85.84-99.93)	15.79 (6.02-31.25)	0.738 (0.626-0.850)
**Jackson, 2016**	77	Dem: 47 (61.0)	3	97 (87-99)	40 (23-60)	0.91 (0.83-0.98)
			7	83 (69-92)	90 (72-97)	

**Taylor-Rowan, 2022**	102	Dem: 12 (11.8)	2	92.3 (64.7-99.8)	58.2 (47.4-68.5)	0.87 (0.79-0.93)
			4	90.9 (58.7-99.8)	80.7 (71.1-88.1)	
Participant AD8						
Community/primary-care setting						
**Cai, 2021**	523	Dem: 17 (3.2)	1	100	41.6	0.931 (0.886-0.976)
			2	100	72.0	
			3	70.6	89.0	
**Dong, 2021**	4481<br><br>DTA: 4463	Dem: 139 (3.1)<br><br>DTA: Dem: 137 (3.1)	3	79	80	0.86 (0.82-0.89)
**Kim, 2019**	420	Dem: 170 (40.5)	2	71 (64-78)	75 (68-81)	0.80 (0.76-0.85)
			3	61 (53-68)	89 (83-93)	
**Yang, 2016**	2015	Dem: 444 (22.0)	2	89.64 (87.54-91.24)	78.42 (76.54-80.84)	0.8402 (0.8204-0.8613)
Secondary care/memory clinic setting						
**Mansbach, 2016**	357	Dem: 229 (64.1)	1	91 (86-94)	24 (17-33)	0.62 (0.56-0.68)
			2	74 (70-80)	37 (29-46)	

†**NC: no cognitive impairment (determined by CDR score of 0), Dem: dementia (determined by CDR score of ≥1)**

Abbreviations: AD, Alzheimer's Dementia; AUC, Area under ROC curve; CDR, Clinical Dementia Rating; Dem, Dementia; DTA, Diagnostic Test Accuracy.

The cut-offs used for each index test (informant and participant AD8) to indicate a positive dementia screen are decided by the individual study authors. The creators of the informant AD8 screening tool originally utilize a cut-off of 2 and a cut-off of 1 when using the informant and participant AD8 respectively to indicate a positive dementia screen, as they provided the optimal predictive performance when compared to reference standard testing in those study populations. Cut-off values are greater than or equal to the numbers listed in the tables.

CDR stands for the Clinical Dementia Rating scale which is used to determine the presence and absence of cognitive impairment and stage its severity. A global score of CDR 0 indicates no dementia, CDR 0.5 indicates mild cognitive impairment (MCI), CDR ≥1 indicates dementia

S5 Table. Predictive parameters of AD8 to screen for CI (MCI or Dementia)

Author, year	Sample size (N)	Prevalence of cognitive disorder – n (%)	Cut-off used	Sensitivity % (95% CI)	Specificity % (95% CI)	AUC (95% CI)
			Informant AD8			
Community/primary-care setting						
**Correia, 2011**	109	CI: 32 (29.4) †	3	83.1	68.7	0.861 (0.790-0.930)
**Galvin, 2007**	325	CI: 176 (54.2) †	1	90	68	0.89 (0.86-0.93)
			2	84	93	
			3	76	90	
**Galvin, 2005**	236	CI: 124 (52.5) †	2	85	86	0.904
**Kasai, 2021**	93	CI: 58 (62.4) †	0/1	65.5	54.3	0.66 (0.55-0.77)
			1/2	37.9	88.6	
**Shang, 2021**	160	CI: 66 (41.2)	2	69.70 (57.1-80.4)	59.57 (49.0-69.6)	0.65 (0.57-0.72)
			3	56.06 (43.3-68.3)	75.53 (65.6-83.8)	0.66 (0.58-0.73)
			4	46.97 (34.6-59.7)	83.81 (75.6-92.3)	0.65 (0.57-0.73)
**Yang, 2011**	239	CI: 125 (52.3) †	2	97.6	78.1	0.961
Secondary care/memory clinic setting						
**Carnero Pardo, 2013**	407	CI: 302 (74.2)	3/4	93 (88-96)	81 (72-88)	0.90 (0.86-0.93)
**Chio, 2018**	153	CI: 110 (71.9) †	1	93 (86-97)	33 (19-49)	0.77 (0.69-0.85)
			2	88 (81-94)	37 (23-53)	
			3	82 (73-89)	53 (38-69)	
			4	71 (61-79)	63 (47-77)	
**Dong, 2013**	280	CI: 243 (86.8) †	2	92.6	86.5	0.96 (0.93-0.98)
**Galvin, 2006**	255<br><br>CDR available for 250	CI: 226 (90.4) †	2	92 (88.8-95.8)	46 (28.0-64.9)	0.915 (0.878-0.952)
			3	90 (86.2-94.0)	68 (50.6-85.2)	
**Larner, 2017**	46	CI: 35 (76.1)	2	97	18	
**Ryu, 2009**	155	CI: 115 (74.2) †	2	78	90	0.88 (0.83-0.93)
			3	67	95	

**Usarel,** 2019	334	CI: 178 (53.3)	3	93.82	93.59	0.979 (0.967-0.992)
Tertiary care/hospitalized setting						
**Duggan,** 2020	75	CI: 71 (94.7)†	2	91.55 (82.51-96.83)	25.00 (0.63-80.59)	0.734 (0.538-0.930)
**Taylor-Rowan,** 2022	102	CI: 43 (42.2)	2	87.1 (70.2-96.4)	68.5 (56.6-78.9)	0.84 (0.76-0.90)
			3	73.3 (54.1-87.7)	81.8 (70.3-89.3)	
Participant AD8						
Community/primary-care setting						
**Chin,** 2013	178	CI: 105 (59.0)	1	85	74	0.80 (0.74-0.86)
			2	73	80	
			3	59	85	
**Galvin,** 2007	325	CI: 176 (54.2)†	1	80	59	0.74 (0.68-0.79)
			2	62	73	
			3	47	82	
**Kasai,** 2021	93	CI: 58 (62.4) †	0/1	85.5	45.7	0.74 (0.64-0.84)
			1/2	70.7	65.7	
Secondary care/memory clinic setting						
**Chio,** 2018	153	CI: 110 (71.9) †	1	81 (72-88)	28 (15-44)	0.59 (0.49-0.69)
			2	55 (45-64)	63 (47-77)	
			3	32 (23-41)	79 (64-90)	
			4	16 (10-25)	86 (73-95)	
**Dong,** 2013	280	CI: 243 (86.8) †	2	59.3	64.9	0.66 (0.58-0.74)

†NC: no cognitive impairment (determined by CDR score of 0), CI: cognitive impairment (determined by CDR score of ≥0.5)

Abbreviations: AUC, Area under ROC curve; CI, Cognitive Impairment; CDR, Clinical Dementia Rating.

The cut-offs used for each index test (informant and participant AD8) to indicate a positive dementia screen are decided by the individual study authors. The creators of the informant AD8 screening tool originally utilize a cut-off of 2 and a cut-off of 1 when using the informant and participant AD8 respectively to indicate a positive dementia screen, as they provided the optimal predictive performance when compared to reference standard testing in those study populations. Cut-off values are greater than or equal to the numbers listed in the tables.

CDR stands for the Clinical Dementia Rating scale which is used to determine the presence and absence of cognitive impairment and stage its severity. A global score of CDR 0 indicates no dementia, CDR 0.5 indicates mild cognitive impairment (MCI), CDR ≥1 indicates dementia.

S6 Table. Test characteristics of the various AD8 score cut-offs for informants and participants

AD8 cut-off	No. of studies	N	Sensitivity (%) CI (%)	Specificity (%) CI (%)	PPV (%) CI (%)	NPV (%) CI (%)
**Informant: MCI**						
≥1	3	977	68.6 (62.8-73.9)	71.6 (67.8-75.1)	53.4 (48.2-58.5)	82.8 (79.2-85.9)
≥2	7	1,266	80 (75-84)	79 (75-83)	73 (68-77)	84 (81-88)
≥3	3	598	68 (59.9-75.2)	79.9 (74.5-84.3)	65 (56.9-72.3)	82 (76.7-86.3)
**Participant: MCI**						
≥1	3	1,058	71.6 (65.9-76.7)	44.6 (40-49.3)	45 (40.4-49.7)	71.2 (65.4-76.3)
≥2	4	1,338	57 (52-62)	71 (67-75)	59 (53-64)	70 (65-74)
≥3	3	1,058	38.9 (33.3-44.9)	86.4 (82.8-89.4)	64.5 (56.8-71.6)	69 (65-72.8)
**Informant: Dementia**						
≥1/2	3	9,090	87.4 (84.8-89.7)	84 (83.1-84.7)	33.3 (31.2-35.4)	98.6 (98.3-98.9)
≥3/4	1	309	91 (77.4-97)	91 (86.5-94.1)	64.5 (51.3-76)	98.2 (95.2-99.4)
≥5/6	1	245	90.6 (84.6-94.4)	83.7 (73.9-90.5)	91.1 (85.3-94.9)	82.7 (72.8-89.7)
≥1	1	776	26.6 (15.1-42.2)	53.8 (50.1-57.4)	3.4 (1.8-6.0)	92.2 (89.2-94.5)
≥2	9	2,579	91 (89-93)	64 (62-67)	58 (55-61)	93 (91-94)
≥3	7	2,641	85.6 (82.3-88.3)	79.3 (77.3-81)	55.1 (51.7-58.5)	94.9 (93.6-95.9)
≥4	4	1,376	89.1 (84.8-92.4)	96.9 (95.6-97.8)	88.5 (84.1-91.8)	97.1 (95.8-98)
≥5	1	334	100 (96-100)	96.2 (91.4-98.4)	95.1 (89.3-98)	100 (96.9-100)
≥7	1	77	83 (68.7-91.9)	87.5 (60.4-97.8)	95.1 (82.2-99.2)	63.6 (40.8-82)
**Participant: Dementia**						
≥1	2	880	91.5 (87.1-94.5)	36.9 (32.6-41.5)	42.9 (38.7-47.3)	89.3 (84-93.1)
≥2	4	3,315	82 (79-84)	75 (73-77)	55 (53-58)	55 (53-58)
≥3	3	5,406	69.1 (63.7-74.1)	80.4 (79.2-81.5)	19 (16.8-21.4)	97.5 (97-98)
**Informant: CI (MCI or Dementia)**						
≥0/1	1	93	65.5 (51.8-77.2)	54.3 (36.9-70.8)	70.4 (56.2-81.6)	48.7 (32.7-65)
≥1/2	1	93	37.9 (25.8-51.7)	88.6 (72.3-96.3)	84.6 (64.3-95)	46.3 (34.2-58.8)
≥3/4	1	407	93.1 (89.4-95.5)	81 (71.9-87.7)	93.4 (89.8-95.8)	80.2 (71.1-87.1)
≥1	2	478	90.9 (86.8-93.9)	59.9 (52.6-66.8)	77.1 (72.2-81.4)	81.6 (74-87.4)
≥2	11	2,056	88 (86-90)	75 (72-79)	87 (86-89)	77 (73-80)
≥3	8	1,588	81.1 (78.4-83.5)	82.4 (79.2-85.2)	87.2 (84.7-89.3)	74.7 (71.3-77.8)
≥4	2	313	61.9(54.3-69)	77.4 (69.3-83.9)	77.9 (70-84.2)	61.3 (53.6-68.5)
**Participant: CI (MCI or Dementia)**						
≥0/1	1	93	86.2 (74-93.4)	45.7 (29.2-63.1)	72.5 (60.2-82.2)	66.7 (44.7-83.6)
≥1/2	1	93	70.7 (57.1-81.5)	65.7 (47.7-80.3)	77.4 (63.5-87.3)	57.5 (41-72.6)
≥1	3	656	81.6 (77.3-85.2)	58.1 (51.9-64)	74.2 (69.7-78.2)	68.1 (61.6-74.1)
≥2	4	936	62 (58-65)	72 (67-77)	82 (78-86)	47 (43-52)
≥3	3	656	46 (41-51.1)	82.3 (77-86.6)	79.3 (73.3-84.3)	50.8 (46-55.6)
≥4	1	153	16.4 (10.2-24.9)	86 (71.4-94.2)	75 (52.9-89.4)	28.7 (21.2-37.4)

CI: Confidence interval; MCI: Mild cognitive impairment; NPV: Negative predictive value; PPV: Positive predictive value

S7 Table. Search Strategy

Medline

Ovid MEDLINE(R) 1946 to March 25, 2022

#	Searches	Results
1	"ascertain dementia 8*".mp.	20
2	"ascertain dementia eight*".mp.	0
3	("AD8" and (dement* or cognit* or alzheimer* or neurocogni* or neuropsych* or psychomet*)).mp.	89
4	("AD-8" and (dement* or cognit* or alzheimer* or neurocog* or neuropsych* or psychomet*)).mp.	48
5	("AD8i" and (dement* or cognit* or alzheimer* or neurocogni* or neuropsych* or psychomet*)).mp.	0
6	("AD-8i" and (dement* or cognit* or alzheimer* or neurocogni* or neuropsych* or psychomet*)).mp.	1
7	(("8-item" adj3 (informant* or participant* or interview? or self-rat* or selfrat*)) and (dement* or cognit* or alzheimer* or neurocog* or neuropsych* or psychomet*)).mp.	11
8	(("eight-item" adj3 (informant* or participant* or interview? or self-rat* or selfrat*)) and (dement* or cognit* or alzheimer* or neurocog* or neuropsych* or psychomet*)).mp.	13
9	or/1-8 [AD8 - Ascertain Dementia 8-item]	156
10	limit 9 to english language	147
11	limit 10 to yr="2004 -Current"	133
12	remove duplicates from 11	132
13	limit 12 to ("all infant (birth to 23 months)" or "all child (0 to 18 years)" or "newborn infant (birth to 1 month)" or "infant (1 to 23 months)" or "preschool child (2 to 5 years)" or "child (6 to 12 years)" or "adolescent (13 to 18 years)" or "young adult (19 to 24 years)" or "adult (19 to 44 years)")	22
14	12 not 13	110
15	limit 12 to ("all adult (19 plus years)" or "middle age (45 to 64 years)" or "middle aged (45 plus years)" or "all aged (65 and over)" or "aged (80 and over)")	121
16	14 or 15 [AD8 with limits applied]	132

Medline In-Process

Ovid MEDLINE(R) Epub Ahead of Print and In-Process, In-Data-Review & Other Non-Indexed Citations March 25, 2022

#	Searches	Results
1	"ascertain dementia 8*".mp.	4
2	"ascertain dementia eight*".mp.	2
3	("AD8" and (dement* or cognit* or alzheimer* or neurocogni* or neuropsych* or psychomet*)).mp.	29
4	("AD-8" and (dement* or cognit* or alzheimer* or neurocog* or neuropsych* or psychomet*)).mp.	5
5	("AD8i" and (dement* or cognit* or alzheimer* or neurocogni* or neuropsych* or psychomet*)).mp.	0
6	("AD-8i" and (dement* or cognit* or alzheimer* or neurocogni* or neuropsych* or psychomet*)).mp.	0
7	(("8-item" adj3 (informant* or participant* or interview? or self-rat* or selfrat*)) and (dement* or cognit* or alzheimer* or neurocog* or neuropsych* or psychomet*)).mp.	3
8	(("eight-item" adj3 (informant* or participant* or interview? or self-rat* or selfrat*)) and (dement* or cognit* or alzheimer* or neurocog* or neuropsych* or psychomet*)).mp.	5
9	or/1-8 [AD8 - Ascertain Dementia 8-item]	34
10	limit 9 to english language	34
11	limit 10 to yr="2004 -Current"	34
12	remove duplicates from 11	34

13	12 not (adolescence or adolescent or adolescents or babies or baby or boy or boys or child or childhood or children or childrens or children's or fetus or fetal or foetus or foetal or girl or girls or infancy or infant or infants or neonatal or neonatally or neonate or neonates or newborn or newborns or paediatric or paediatrician or paediatricians or paediatrics or pediatric or pediatrician or pediatricians or pediatrics or preschool* or teen or teenage or teenagers or teens or toddler or toddlers or tween* or youth or youths).ti,jw.	32
14	12 and (elder* or senior? or aged or adult* or man or men or woman* or women).ti,jw,hw.	12
15	13 or 14	33
16	12 not 15 [checked citation – adult]	1
17	15 or 16	34

Embase
Embase Classic+Embase 1947 to 2022 March 25

#	Searches	Results
1	"ascertain dementia 8*".mp.	36
2	"ascertain dementia eight*".mp.	0
3	("AD8" and (dement* or cognit* or alzheimer* or neurocogni* or neuropsych* or psychomet*)).mp.	208
4	("AD-8" and (dement* or cognit* or alzheimer* or neurocog* or neuropsych* or psychomet*)).mp.	117
5	("AD8i" and (dement* or cognit* or alzheimer* or neurocogni* or neuropsych* or psychomet*)).mp.	0
6	("AD-8i" and (dement* or cognit* or alzheimer* or neurocogni* or neuropsych* or psychomet*)).mp.	1
7	(("8-item" adj3 (informant* or participant* or interview? or self-rat* or selfrat*)) and (dement* or cognit* or alzheimer* or neurocog* or neuropsych* or psychomet*)).mp.	19
8	(("eight-item" adj3 (informant* or participant* or interview? or self-rat* or selfrat*)) and (dement* or cognit* or alzheimer* or neurocog* or neuropsych* or psychomet*)).mp.	18
9	or/1-8 [AD8 - Ascertain Dementia 8-item]	345
10	limit 9 to english language	335
11	limit 10 to yr="2004 -Current"	318
12	remove duplicates from 11	314
13	limit 12 to (conference abstracts or "preprints (unpublished, non-peer reviewed)" or (books or chapter or conference abstract or conference paper or "conference review" or "preprint (unpublished, non-peer reviewed)") or (book or book series or conference proceeding or "preprint archive (unpublished, non-peer reviewed)" or trade journal))	144
14	12 not 13	170
15	14 not (adolescence or adolescent or adolescents or babies or baby or boy or boys or child or childhood or children or childrens or children's or fetus or fetal or foetus or foetal or girl or girls or infancy or infant or infants or neonatal or neonatally or neonate or neonates or newborn or newborns or paediatric or paediatrician or paediatricians or paediatrics or pediatric or pediatrician or pediatricians or pediatrics or preschool* or teen or teenage or teenagers or teens or toddler or toddlers or tween* or youth or youths).ti,jw.	169
16	14 and (elder* or senior? or aged or adult* or man or men or woman* or women).ti,jw.	33
17	15 or 16	170

CCTR
EBM Reviews - Cochrane Central Register of Controlled Trials January 2022

#	Searches	Results
1	"ascertain dementia 8*".mp.	4
2	"ascertain dementia eight*".mp.	0
3	("AD8" and (dement* or cognit* or alzheimer* or neurocogni* or neuropsych* or psychomet*)).mp.	12

4	("AD-8" and (dement* or cognit* or alzheimer* or neurocog* or neuropsych* or psychomet*)).mp.	13
5	("AD8i" and (dement* or cognit* or alzheimer* or neurocogni* or neuropsych* or psychomet*)).mp.	0
6	("AD-8i" and (dement* or cognit* or alzheimer* or neurocogni* or neuropsych* or psychomet*)).mp.	0
7	(("8-item" adj3 (informant* or participant* or interview? or self-rat* or selfrat*)) and (dement* or cognit* or alzheimer* or neurocog* or neuropsych* or psychomet*)).mp.	6
8	(("eight-item" adj3 (informant* or participant* or interview? or self-rat* or selfrat*)) and (dement* or cognit* or alzheimer* or neurocog* or neuropsych* or psychomet*)).mp.	2
9	or/1-8 [AD8 - Ascertain Dementia 8-item]	31
10	limit 9 to english language	19
11	limit 10 to yr="2004 -Current"	17
12	remove duplicates from 11	16
13	12 not (abstract or addresses or bibliography or biography or book or book article or book book or book note or "book review" or book series article or book series article in press or book series chapter or book series conference paper or book series letter or "book series review" or book series short survey or chapter or conference or conference abstract or conference abstract placebo controlled partly blinded crossover study in 12 sle patients or conference proceeding or "conference review" or journal conference abstract or "journal conference review" or monograph or conferenc* or book*).pt.	11

CDSR
EBM Reviews - Cochrane Database of Systematic Reviews 2005 to March 24, 2022

#	Searches	Results
1	"ascertain dementia 8*".mp.	1
2	"ascertain dementia eight*".mp.	0
3	("AD8" and (dement* or cognit* or alzheimer* or neurocogni* or neuropsych* or psychomet*)).mp.	8
4	("AD-8" and (dement* or cognit* or alzheimer* or neurocog* or neuropsych* or psychomet*)).mp.	7
5	("AD8i" and (dement* or cognit* or alzheimer* or neurocogni* or neuropsych* or psychomet*)).mp.	0
6	("AD-8i" and (dement* or cognit* or alzheimer* or neurocogni* or neuropsych* or psychomet*)).mp.	0
7	(("8-item" adj3 (informant* or participant* or interview? or self-rat* or selfrat*)) and (dement* or cognit* or alzheimer* or neurocog* or neuropsych* or psychomet*)).mp.	1
8	(("eight-item" adj3 (informant* or participant* or interview? or self-rat* or selfrat*)) and (dement* or cognit* or alzheimer* or neurocog* or neuropsych* or psychomet*)).mp.	2
9	or/1-8 [AD8 - Ascertain Dementia 8-item]	15
10	limit 9 to full systematic reviews	14

APA PsycINFO
APA PsycInfo 1806 to March Week 3 2022

#	Searches	Results
1	"ascertain dementia 8*".mp.	15
2	"ascertain dementia eight*".mp.	2
3	("AD8" adj10 (dement* or cognit* or alzheimer* or neurocogni* or neuropsych* or psychomet*)).mp.	63
4	("AD-8" adj10 (dement* or cognit* or alzheimer* or neurocog* or neuropsych* or psychomet*)).mp.	25
5	(("8-item" adj3 (informant* or participant* or interview? or self-rat* or selfrat*)) and (dement* or cognit* or alzheimer* or neurocog* or neuropsych* or psychomet*)).mp.	13
6	(("eight-item" adj3 (informant* or participant* or interview? or self-rat* or selfrat*)) and (dement* or cognit* or alzheimer* or neurocog* or neuropsych* or psychomet*)).mp.	12

7	Ascertain Dementia*.tm.	16
8	or/1-7 [AD8 - Ascertain Dementia 8-item]	112
9	remove duplicates from 8	112
10	limit 9 to english language	106
11	limit 10 to yr="2004 -Current"	96
12	limit 11 to ("0200 book" or "0240 authored book" or "0280 edited book" or "0300 encyclopedia" or "0400 dissertation abstract" or (abstract collection or bibliography or chapter or dissertation or encyclopedia entry or obituary or poetry or review-book or review-media or review-software & other) or (classic book or conference proceedings or "handbook/manual" or reference book or "textbook/study guide"))	2
13	11 not 12	94
14	13 not (adolescence or adolescent or adolescents or babies or baby or boy or boys or child or childhood or children or childrens or children's or fetus or fetal or foetus or foetal or girl or girls or infancy or infant or infants or neonatal or neonatally or neonate or neonates or newborn or newborns or paediatric or paediatrician or paediatricians or paediatrics or pediatric or pediatrician or pediatricians or pediatrics or preschool* or teen or teenage or teenagers or teens or toddler or toddlers or tween* or youth or youths).ti,jw.	93
15	13 and (elder* or senior? or aged or adult* or man or men or woman* or women).ti,jw.	14
16	14 or 15	93

Ovid Emcare Nursing
Ovid Emcare Nursing 1995 to Present

#	Searches	Results
1	"ascertain dementia 8*".mp.	17
2	"ascertain dementia eight*".mp.	0
3	("AD8" and (dement* or cognit* or alzheimer* or neurocogni* or neuropsych* or psychomet*)).mp.	68
4	("AD-8" and (dement* or cognit* or alzheimer* or neurocog* or neuropsych* or psychomet*)).mp.	32
5	("AD8i" and (dement* or cognit* or alzheimer* or neurocogni* or neuropsych* or psychomet*)).mp.	0
6	("AD-8i" and (dement* or cognit* or alzheimer* or neurocogni* or neuropsych* or psychomet*)).mp.	0
7	(("8-item" adj3 (informant* or participant* or interview? or self-rat* or selfrat*)) and (dement* or cognit* or alzheimer* or neurocog* or neuropsych* or psychomet*)).mp.	7
8	(("eight-item" adj3 (informant* or participant* or interview? or self-rat* or selfrat*)) and (dement* or cognit* or alzheimer* or neurocog* or neuropsych* or psychomet*)).mp.	8
9	or/1-8 [AD8 - Ascertain Dementia 8-item]	113
10	limit 9 to english language	112
11	limit 10 to yr="2004 -Current"	107
12	remove duplicates from 11	107
13	limit 12 to (books or business article or chapter or conference abstract or conference paper or "conference review" or dissertation or press release or (book or book series or conference proceeding or major reference work or report or trade journal))	6
14	12 not 13	101
15	14 not (exp animals/ or exp animal experimentation/ or nonhuman/) not ((exp animals/ or exp animal experimentation/ or nonhuman/) and exp human/)	8
16	limit 14 to human	93
17	15 or 16	101
18	limit 17 to (embryo <first trimester> or infant <to one year> or child <unspecified age> or preschool child <1 to 6 years> or school child <7 to 12 years> or adolescent <13 to 17 years>)	3
19	17 not 18	98

20	limit 17 to (adult <18 to 64 years> or aged <65+ years>)	79
21	19 or 20	101

Ovid Journals@Ovid
OVFT full text search. Limited to human, aged adults. NB: NOT ABLE TO LIMIT TO ENGLISH.

#	Searches	Results
1	"ascertain dementia 8*".mp.	43
2	"ascertain dementia eight*".mp.	2
3	("AD8" adj10 (dement* or cognit* or alzheimer* or neurocogni* or neuropsych* or psychomet*)).mp.	288
4	("AD-8" adj10 (dement* or cognit* or alzheimer* or neurocog* or neuropsych* or psychomet*)).mp.	552
5	("AD8i" adj10 (dement* or cognit* or alzheimer* or neurocogni* or neuropsych* or psychomet*)).mp.	0
6	("AD-8i" adj10 (dement* or cognit* or alzheimer* or neurocogni* or neuropsych* or psychomet*)).mp.	0
7	(("8-item" adj3 (informant* or participant* or interview? or self-rat* or selfrat*)) and (dement* or cognit* or alzheimer* or neurocog* or neuropsych* or psychomet*)).mp.	191
8	(("eight-item" adj3 (informant* or participant* or interview? or self-rat* or selfrat*)) and (dement* or cognit* or alzheimer* or neurocog* or neuropsych* or psychomet*)).mp.	220
9	or/1-8 [AD8 - Ascertain Dementia 8-item]	1199
10	limit 9 to yr="2004 -Current"	1085
11	limit 10 to (advertisements or announcements or "book or media reviews" or meeting abstracts or reports)	86
12	10 not 11	999
13	remove duplicates from 12	992
14	13 not (animal or animals or ape or apes or baboon or baboons or bat or bats or beagle or beagles or bird or birds or boar or boars or bonobo or bonobos or bovine or camel or camels or canine or canines or cat or cats or cattle or chick or chicks or chicken or chickens or chimpanzee or chimpanzees or dog or dogs or dromedary or dromedaries or duck or ducks or equine or equines or feline or felines or ferret or ferrets or frog or frogs or fowl or fowls or goat or goats or hamster or hamsters or hare or hares or hen or hens or horse or horses or lamb or lambs or livestock or macaque or macaques or mandrill or mandrills or mice or mink or minks or monkey or monkeys or mouse or murine or ovine or pig or pigs or piglet or piglets or poultry or porcine or orangutan or orangutans or rabbit or rabbits or rat or rats or rodent or rodents or sheep or swine or tamarin or tamarins or tiger or tigers or veterinary or veterinarian or veterinarians or waterfowl or waterfowls or weasel or weasels or veterinar* or fish or shellfish).ti,jw.	959
15	"older than 1##".mp.	3825
16	"older than 6#".mp.	45475
17	"older than 7#".mp.	20545
18	"older than 8#".mp.	9942
19	"older than 9#".mp.	1671
20	("over 1##" adj8 year?).mp.	1064560
21	("over 6#" adj8 year?).mp.	962544
22	("over 7#" adj8 year?).mp.	690747
23	("over 8#" adj8 year?).mp.	493064
24	("over 9#" adj8 year?).mp.	336333
25	((old?? or advance?) adj (age or aging or ageing)).mp.	248319
26	((old?? or elder?? or senior?) adj (patient? or citizen?? or person? or people or geriatric* or population?)).mp.	459786
27	(aged adj2 "10# years").mp.	2186
28	(aged adj2 "6# years").mp.	104524
29	(aged adj2 "65 years").mp.	55389

30	(aged adj2 "7# years").mp.	61473
31	(aged adj2 "8# years").mp.	34953
32	(aged adj2 "9# years").mp.	8972
33	(elder* adj1 patient?).mp.	153805
34	(old adj age).mp.	63569
35	(old* adj1 patient?).mp.	245933
36	(older adult* or older client* or older patient* or older person* or older people).mp.	316840
37	centenarian*.mp.	3177
38	elder?.mp.	49908
39	elderly.mp.	426061
40	geriatri*.mp.	115937
41	grandfather*.mp.	12724
42	grandma??.mp.	1419
43	grandmother*.mp.	18649
44	grandpa??.mp.	588
45	grandparent*.mp.	21300
46	nonagenarian*.mp.	2153
47	octagenarian*.mp.	98
48	octogenarian*.mp.	6436
49	oncogeriatri*.mp.	155
50	onco-geriatri*.mp.	32
51	orthogeriatri*.mp.	766
52	ortho-geriatri*.mp.	94
53	postmaturity.mp.	363
54	post-maturity.mp.	153
55	psychogeriatri*.mp.	3020
56	psycho-geriatri*.mp.	238
57	retiree*.mp.	5946
58	retirement?.mp.	52338
59	senior citizen*.mp.	5171
60	septuagenarian*.mp.	849
61	sexagenarian*.mp.	148
62	supercentenarian*.mp.	145
63	super-centenarian*.mp.	31
64	"6# year?".mp.	642001
65	"7# year?".mp.	420071
66	"8# year?".mp.	258321
67	"9# year?".mp.	106838
68	"10# year?".mp.	90008
69	"age? 6#".mp.	332672
70	"age? 7#".mp.	147178
71	"age? 8#".mp.	66941

72	"age? 9#".mp.	21900
73	"age? 10#".mp.	18452
74	"extreme age?".mp.	1447
75	(oldest adj2 old?).mp.	7145
76	(older adj2 adult?).mp.	166316
77	or/15-76	2660457
78	14 and 77	786
79	78 not (adolescence or adolescent or adolescents or babies or baby or boy or boys or child or childhood or children or childrens or children's or fetus or fetal or foetus or foetal or girl or girls or infancy or infant or infants or neonatal or neonatally or neonate or neonates or newborn or newborns or paediatric or paediatrician or paediatricians or paediatrics or pediatric or pediatrician or pediatricians or pediatrics or preschool* or teen or teenage or teenagers or teens or toddler or toddlers or tween* or youth or youths).ti,jw.	766
80	article.pt.	3871183
81	79 and 80	489

Web of Science
Data updated 2022-03-27

TS=("ascertain dementia 8*" OR
"ascertain dementia eight*" OR
(AD8 AND (dement* OR cognit* OR alzheimer* OR neurocogni* OR neuropsych* OR psychomet*)) OR
(AD-8 AND (dement* OR cognit* OR alzheimer* OR neurocog* OR neuropsych* OR psychomet*)) OR
(AD8i AND (dement* OR cognit* OR alzheimer* OR neurocogni* OR neuropsych* OR psychomet*)) OR
(AD-8i AND (dement* OR cognit* OR alzheimer* OR neurocogni* OR neuropsych* OR psychomet*)) OR
((8-item NEAR/3 (informant* OR participant* OR interview$ OR self-rat* OR selfrat*)) AND (dement* OR cognit* OR alzheimer* OR neurocog* OR neuropsych* OR psychomet*)) OR
((eight-item NEAR/3 (informant* OR participant* OR interview$ OR self-rat* OR selfrat*)) AND (dement* OR cognit* OR alzheimer* OR neurocog* OR neuropsych* OR psychomet*)) OR
("ascertain dementia 8*") OR ("ascertain dementia eight*") OR ((AD8 AND (dement* OR cognit* OR alzheimer* OR neurocogni* OR neuropsych* OR psychomet*))) OR ((AD-8 AND (dement* OR cognit* OR alzheimer* OR neurocog* OR neuropsych* OR psychomet*))) OR ((AD8i AND (dement* OR cognit* OR alzheimer* OR neurocogni* OR neuropsych* OR psychomet*))) OR ((AD-8i AND (dement* OR cognit* OR alzheimer* OR neurocogni* OR neuropsych* OR psychomet*))) OR (((8-item NEAR/3 (informant* OR participant* OR interview$ OR self-rat* OR selfrat*)) AND (dement* OR cognit* OR alzheimer* OR neurocog* OR neuropsych* OR psychomet*))) OR (((eight-item NEAR/3 (informant* OR participant* OR interview$ OR self-rat* OR selfrat*)) AND (dement* OR cognit* OR alzheimer* OR neurocog* OR neuropsych* OR psychomet*)))) and **Articles** or **Early Access** (Document Types) and **English** (Languages) and **10TH ANNUAL ACADEMIC EMERGENCY MEDICINE CONSENSUS CONFERENCE ANNUAL MEETING OF THE SOCIETY FOR ACADEMIC EMERGENCY MEDICINE** or **40TH ANNUAL SCIENTIFIC MEETING OF THE SOCIETY FOR ACADEMIC EMERGENCY MEDICINE SAEM** or **4TH INTERNATIONAL CONGRESS ON VASCULAR DEMENTIA** or **60TH ANNUAL SCIENTIFIC MEETING OF THE GERONTOLOGICAL SOCIETY OF AMERICA** or **74TH ANNUAL MEETING OF THE AMERICAN ASSOCIATION FOR THE SURGERY OF TRAUMA AAST CLINICAL CONGRESS OF ACUTE CARE SURGERY** or **WORLD CONGRESS OF THE INTERNATIONAL ASSOCIATION OF GERONTOLOGY AND GERIATRICS IAGG** (Exclude – Conference Titles) Timespan: 2004-01-01 to 2023-01-01 (Index Date)
192

Scopus
46 document results
(TITLE-ABS-KEY (*"ascertain dementia 8*"*) OR TITLE-ABS-KEY (*"ascertain dementia eight*"*) OR TITLE-ABS-KEY (*ad8* AND (*dement** OR *cognit** OR *alzheimer** OR *neurocogni** OR *neuropsych** OR *psychomet**)) OR TITLE-ABS-KEY (*ad-8* AND (*dement** OR *cognit** OR *alzheimer** OR *neurocog** OR *neuropsych** OR *psychomet**)) OR TITLE-ABS-KEY (*ad8i* AND (*dement** OR *cognit** OR *alzheimer** OR *neurocogni** OR *neuropsych** OR *psychomet**)) OR TITLE-ABS-KEY (*ad-8i* AND (*dement** OR *cognit** OR *alzheimer** OR *neurocogni** OR *neuropsych** OR *psychomet**)) OR TITLE-ABS-KEY ((*8-item* W/3 (*informant** OR *participant** OR *interview** OR *self-*

*rat** OR *selfrat**)) AND (*dement** OR *cognit** OR *alzheimer** OR *neurocog** OR *neuropsych** OR *psychomet**)) OR TITLE-ABS-KEY ((*eight-item* W/3 (*informant** OR *participant** OR *interview** OR *self-rat** OR *selfrat**)) AND (*dement** OR *cognit** OR *alzheimer** OR *neurocog** OR *neuropsych** OR *psychomet**)) OR (TITLE-ABS-KEY (*"ascertain dementia 8*"*)) OR (TITLE-ABS-KEY (*"ascertain dementia eight*"*)) OR (TITLE-ABS-KEY (*ad8* AND (*dement** OR *cognit** OR *alzheimer** OR *neurocogni** OR *neuropsych** OR *psychomet**))) OR (TITLE-ABS-KEY (*ad-8* AND (*dement** OR *cognit** OR *alzheimer** OR *neurocog** OR *neuropsych** OR *psychomet**))) OR (TITLE-ABS-KEY (*ad8i* AND (*dement** OR *cognit** OR *alzheimer** OR *neurocogni** OR *neuropsych** OR *psychomet**))) OR (TITLE-ABS-KEY (*ad-8i* AND (*dement** OR *cognit** OR *alzheimer** OR *neurocogni** OR *neuropsych** OR *psychomet**))) OR (TITLE-ABS-KEY ((*8-item* W/3 (*informant** OR *participant** OR *interview** OR *self-rat** OR *selfrat**)) AND (*dement** OR *cognit** OR *alzheimer** OR *neurocog** OR *neuropsych** OR *psychomet**))) OR (TITLE-ABS-KEY ((*eight-item* W/3 (*informant** OR *participant** OR *interview** OR *self-rat** OR *selfrat**)) AND (*dement** OR *cognit** OR *alzheimer** OR *neurocog** OR *neuropsych** OR *psychomet**))))) AND NOT (INDEX (*medline*)) AND (LIMIT-TO (DOCTYPE , *"ar"*)) AND (EXCLUDE (PUBYEAR , *2002*) OR EXCLUDE (PUBYEAR , *2001*) OR EXCLUDE (PUBYEAR , *2000*) OR EXCLUDE (PUBYEAR , *1999*) OR EXCLUDE (PUBYEAR , *1996*) OR EXCLUDE (PUBYEAR , *1995*) OR EXCLUDE (PUBYEAR , *1991*) OR EXCLUDE (PUBYEAR , *1990*)) AND (LIMIT-TO (LANGUAGE , *"English"*))

CINAHL

AD8 - Ascertain Dementia 8-item - BROAD SR search w limits

#	Query	Limiters/Expanders	Last Run Via	Results
S1	TX "ascertain dementia 8*" OR "ascertain dementia eight*" OR (AD8 AND (dement* OR cognit* OR alzheimer* OR neurocogni* OR neuropsych* OR psychomet*)) OR (AD-8 AND (dement* OR cognit* OR alzheimer* OR neurocog* OR neuropsych* OR psychomet*)) OR (AD8i AND (dement* OR cognit* OR alzheimer* OR neurocogni* OR neuropsych* OR psychomet*)) OR (AD-8i AND (dement* OR cognit* OR alzheimer* OR neurocogni* OR neuropsych* OR psychomet*)) OR ((8-item N3 (informant* OR participant* OR interview# OR self-rat* OR selfrat*)) AND (dement* OR cognit* OR alzheimer* OR neurocog* OR neuropsych* OR psychomet*)) OR ((eight-item N3 (informant* OR participant* OR interview# OR self-rat* OR selfrat*)) AND (dement* OR cognit* OR alzheimer* OR neurocog* OR neuropsych* OR psychomet*)) OR ("ascertain dementia 8*") OR ("ascertain dementia eight*") OR ((AD8 AND (dement* OR cognit* OR alzheimer* OR neurocogni* OR neuropsych* OR psychomet*))) OR ((AD-8 AND (dement* OR cognit* OR alzheimer* OR neurocog* OR neuropsych* OR psychomet*))) OR ((AD8i AND (dement* OR cognit* OR alzheimer* OR neurocogni* OR neuropsych* OR psychomet*))) OR ((AD-8i AND (dement* OR cognit* OR alzheimer* OR neurocogni* OR neuropsych* OR psychomet*))) OR (((8-item N3 (informant* OR participant* OR interview# OR self-rat* OR selfrat*)) AND (dement* OR cognit* OR alzheimer* OR neurocog* OR neuropsych* OR psychomet*))) OR (((eight-item N3 (informant* OR participant* OR interview# OR self-rat* OR selfrat*)) AND (dement* OR cognit* OR alzheimer* OR neurocog* OR neuropsych* OR psychomet*)))	Limiters - Published Date: 20040101-20231231; English Language; Peer Reviewed; Exclude MEDLINE records; Age Groups: Middle Aged: 45-64 years, Aged: 65+ years, Aged, 80 and over, All Adult<br>Expanders - Apply equivalent subjects<br>Search modes - Boolean/Phrase	Interface - EBSCOhost Research Databases<br>Search Screen - Advanced Search<br>Database - CINAHL Complete	122

Web of Science CITATION SEARCHING

Data updated 2022-03-27

Combined citing = 633

Citing Results: Citations of The AD8 - A brief informant interview to detect dementia

1 The AD8 - A brief informant interview to detect dementia

Galvin, JE; Roe, CM; (...); Morris, JC
Aug 23 2005 | NEUROLOGY 65 (4) , pp.559-564

Background: Brief measures that accurately discriminate normal cognitive aging from very mild dementia are lacking. Cognitive tests often are insensitive to very mild dementia. Informant-based measures may be more sensitive in detecting early dementia. Objective: To identify informant-reported clinical variables that differentiate cognitively normal individuals from those with very mild dementia. Methods: A 55-item battery of info ... Show more

Get it! UTL Full Text at Publisher •••

502 Citations

37 References

Related records

Citing Results: Citations of Validity and reliability of the AD8 informant interview

Validity and reliability of the AD8 informant interview in dementia

Galvin, JE; Roe, CM; (...); Morris, JC
Dec 12 2006 | NEUROLOGY 67 (11) , pp.1942-1948

Objective: To establish the validity, reliability, and discriminative properties of the AD8, a brief informant interview to detect dementia, in a clinic sample. Methods: We evaluated 255 patient-informant dyads. We compared the number of endorsed AD8 items with an independently derived Clinical Dementia Rating (CDR) and with performance on neuropsychological tests. Construct and concurrent validity, test-retest, interrater and int ... Show more

221 Citations

38 References

Citing Results: Citations of Patient's rating of cognitive ability - Using the AD8, a brief informant interview, as a self-rating tool to detect dementia

Patient's rating of cognitive ability - Using the AD8, a brief informant interview, as a self-rating tool to detect dementia

Galvin, JE; Roe, CM; (...); Morris, JC
May 2007 | ARCHIVES OF NEUROLOGY 64 (5) , pp.725-730

Objective: To test the ability of patients to rate their own cognitive ability using the AD8 compared with informant and clinician ratings of cognitive status. Design, Setting, and Patients: The AD8 was administered to 325 consecutive participant-informant dyads enrolled in a longitudinal study at Washington University School of Medicine between April 4, 2005, and December 15, 2005. The number of AD8 items endorsed by the participa ... Show more

97 Citations

39 References

Scopus CITATION SEARCHING

Combined citing = 694

1.	Ascertain Dementia 8-items - 3 articles being cited	694	28 Mar 2022

1

Document title	Authors	Year	Source	Cited by
The AD8: A brief informant interview to detect dementia	Galvin, J.E., Roe, C.M., Powlishta, K.K., (...), Storandt, M., Morris, J.C.	2005	Neurology 65(4), pp. 559-564	554

554 documents have cited:
The AD8: A brief informant interview to detect dementia
Galvin J.E., Roe C.M., Powlishta K.K., Coats M.A., Muich S.J., Grant E., Miller J.P., (...), Morris J.C.
(2005) Neurology, 65 (4) , pp. 559-564.

2

Document title	Authors	Year	Source	Cited by
Validity and reliability of the AD8 informant interview in dementia	Galvin, J.E., Roe, C.M., Xiong, C., Morris, J.C.	2006	Neurology 67(11), pp. 1942-1948	242

242 documents have cited:
Validity and reliability of the AD8 informant interview in dementia
Galvin J.E., Roe C.M., Xiong C., Morris J.C.
(2006) Neurology, 67 (11) , pp. 1942-1948.

3

Document title	Authors	Year	Source	Cited by
Patient's rating of cognitive ability: Using the AD8, a brief informant interview, as a self-rating tool to detect dementia *Open Access*	Galvin, J.E., Roe, C.M., Coats, M.A., Morris, J.C.	2007	Archives of Neurology 64(5), pp. 725-730	113

113 documents have cited:
Patient's rating of cognitive ability: Using the AD8, a brief informant interview, as a self-rating tool to detect dementia
Galvin J.E., Roe C.M., Coats M.A., Morris J.C.
(2007) Archives of Neurology, 64 (5) , pp. 725-730.
